# Techniques and Processes for the Realization of Electrically Conducting Textile Materials from Intrinsically Conducting Polymers and Their Application Potential

**DOI:** 10.3390/polym12122867

**Published:** 2020-11-30

**Authors:** Toty Onggar, Iris Kruppke, Chokri Cherif

**Affiliations:** Institute of Textile Machinery and High Performance Material Technology, Technical University Dresden, 01062 Dresden, Germany; iris.kruppke@tu-dresden.de (I.K.); chokri.cherif@tu-dresden.de (C.C.)

**Keywords:** electrical conducting, intrinsically polymers, textile, spinning, coating

## Abstract

This review will give an overview on functional conducting polymers, while focusing on the integration of intrinsically conducting, i.e., self-conducting, polymers for creating electrically conducting textile materials. Thus, different conduction mechanisms as well as achievable electrical properties will be introduced. First, essential polymers will be described individually, and secondly, techniques and processes for the realization of electrically conducting textile products in addition to their application potential will be presented.

## 1. Introduction

The textile industry has a strong interest in electrically conducting polymers. This is due to their unique combination of properties, offering amongst others, electrical conductivity, electromagnetic shielding, bendability, and stretch ability in addition to the high mechanical flexibility provided by substrates.

Within the last years, there have been different printing technologies developed, such as screen printing, inkjet printing, gravure printing, transfer printing, flexographic printing, and nanoimprinting to apply intrinsically conductive polymers (ICP) onto textile surfaces. The used substrates are natural fibers like cotton (CO), wool (WO), and silk (SE), as well as man-made fibers like polyethylene terephthalate (PET), polypropylene (PP), polyamide (PA), polyacrylonitrile (PAN), and polyurethane (PU). In contrast to this, ICP are achieved using electro spinning, wet spinning, or dry spinning, whereas the spun yarns exhibit a good electrical conductivity but reduced textile mechanical properties. In consequence, the manufacturing of intelligent materials, which consist of electro conductive yarns, is a challenging target. In order to get good mechanical properties with coincident electrical conductivity, coating technologies for textiles and fabrics are a promising alternative. For the application of ICP onto textile materials, two main coating technologies are prevailingly used: dipping-and-drying technique and chemical solution/vapor polymerization.

**Figure 1:**
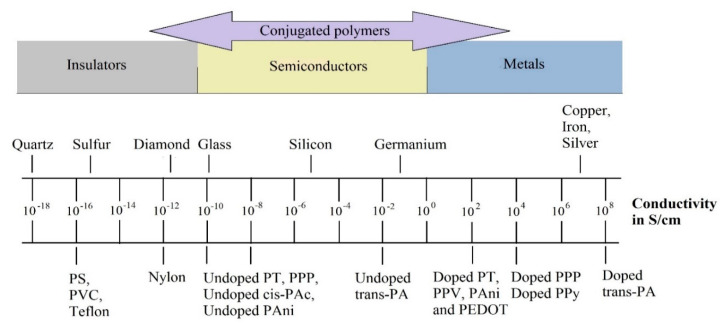
Electrical conductivity of intrinsically conducting polymers compared to other solid materials [1,2,3,4,5].

Intrinsically conducting polymers, so-called conducting polymers, are plastics that are characterized by electrical conductivity and properties that are comparable with metals [1]. The conductivity of polymers is based on conjugated double bonds between atoms enabling the free mobility of doped charge carriers [1]. Electrical conductivity requires freely moving charge carriers; hence, electrically self-conducting polymers have an extensive π-electron system in the form of conjugated double bonds with holes serving as charge carriers. Aromatic or heteroaromatic rings as well as triple bonds also belong to the group of polyconjugated bond systems. The term ICP refers to, for example, polyacetylene (PAc), polypyrrole (PPy), polythiophene (PT), poly(3,4-ethylenedioxythiophene) (PEDOT), polyaniline (PAni), polyselenophene (PSe), polyfuran (PFu), poly(para-phenylene) (PPP), and poly(p-phenylene vinylene) (PPV). Figure 1 illustrates the conductivity of ICPs in comparison to other solid materials [1,2,3,4,5]. The electrical conductivities of these polymer classes are in the range of 10^−13^–10^8^ S/cm in a doped and undoped state (Table 1) [1,2,3,4,5]. These polymers—depending on their state—can act as insulator, semiconductor, or conductor.

In spite of the high interest according to conductive polymers, there are still many challenges in their handling and processing because of the properties of the melt, the susceptibility to moisture as well as the weak stability at high temperatures, the unsolublity in the most solvents, and the instability of oxygen containing gases. Within this review, conductive polymers will be described with their principles of electric conductivity and the achievable electric properties. The processes for the realization of electrical conductive textile products within the assets and drawbacks of the several printing, coating, and spinning techniques will be introduced for the different conductive polymers in accordance to the scope of application. It is described in consideration of the used doping and oxidation materials as well as solvents and other additives, which are used to prepare printing pastes, coatings, or dopes for spinning.

## 2. Electrical Conductivity of ICP

To achieve polymers with a desirable amount of electrical conductivity and stability, they must be fully doped. The electrical conductivity of PAc in a doped state is higher than that of other ICP. In an undoped state, all ICP possess similar levels of electrical conductivity (Table 1). Doping reactions can be subdivided into p-type doping (*p* = positive; oxidation), i.e., a polymer reaction with an oxidizer (acceptor carrying electrons), and n-type doping (*n* = negative; reduction), i.e., a polymer reaction with a reducer (donor carrying electrons) [1,2,3,4,5]. Due to their large number of double bonds, undoped conducting polymers possess high reactivity. Therefore, the cross-linking reactions as a result of oxidation and addition are possible at room temperature. PPP being resistant to oxidation and temperature is an exception to this general rule. However, reactivity plays a minor role in the case of doped polymers due to their oxidized state [1,2].

Although researchers have investigated this topic for at least 40 years, it is still not clear which structural characteristics of (intrinsically) conducting polymers are beneficial or essential in terms of high conductivity. It is generally assumed that high crystallinity and a linear array of chains allow high conductivity, which has been confirmed for technically and commercially relevant polymers, such as PT and PEDOT. In contrast, this assumption could not be confirmed for PAni, which is mostly amorphous and has little to no differences in crystallinity [6]. Other polymers, e.g., PAc and PPP, exhibit a negatively charged polymer structure.

**Figure 2:**
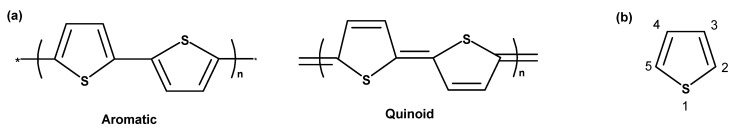
(**a**) Structures of PT and (**b**) thiophene monomer [8,9].

Anions serve as counter ions of the oxidized polymer structure. If electric current flows, charge carriers need to switch from one polymer chain to an adjacent one due to the limited length of conjugated chains—a process termed hopping. Hence, the total resistance results from the sum of resistances within a polymer chain as well as between chains, whereby the higher resistance between chains has a greater influence on electric conductivity. The shorter conjugated chains are, the higher the resulting resistance is, since charge carriers must be transmitted between chains more often [3,4]. Additionally, conductivity is affected by the ageing of polymers and the diffusion path, i.e., decreasing in the case of a shorter diffusion path, which is why thicker polymer films are generally considered to be more stable. Since diffusion is a thermally active process, the corresponding temperature increase is expected to catalyze the loss in conductivity [5]. Another characteristic of ICP is that they offer a particularly stable form of conductivity; in order to guarantee this advantage, monomers and counter ions as well as manufacturing conditions must be selected carefully. Counter ions are introduced for the compensation of free charges generated on chains by oxidation and reduction [7]. The conductivities of PAc and polyheterocycles (PHC) vary widely in terms of their stability: while PAc exhibit a considerable loss in conductivity after 24 h, the conduction properties of PHC remain stable even after several days [6]. The degree of doping affects both conductivity as well as stability. The less doped a polymer is, the lower its stability. The doping and electrical conductivity of polymers can also be enhanced by acids and bases [5,6]. The characteristics of PT are determined by the sulfur heteroatom in the cyclic C–H-bond (Figure 2). In its basic state, PT is not degenerated and can occur in either an aromatic or a quinoid form (Figure 2a). Compared to monosubstituted polymers, disubstituted PT offer a higher potential for oxidation and a wider band gap, however also having lower conductivity. They have a stabilizing effect on the oxidized form of the polymer. Due to cyclization between the 3- and 4-position, steric hindrance can be minimized (Figure 2b). Substituents at this position prevent an α-β-coupling at the 2,5-position during electropolymerization, which typically impairs conductivity and leads to over-oxidation in conducting polymers at high potentials. PEDOT, being a prominent representative of this polymer class, is displayed in Figure 2b [8,9].

Essential parameters for the manufacturing of electrically conducting textile materials include the thorough doping of ICP, high crystallinity, linear arrangement of polymer chains, high resistance between polymer chains, polymer type, low processing temperature, ageing of polymers, short diffusion path, and a high oxidation potential. To understand the relationship between the electrical conductivity of ICP and resulting effects on textile materials, the following chapter will focus on introducing various ICP types.

## 3. Intrinsically Conducting Polymer Types

**Figure 3:**
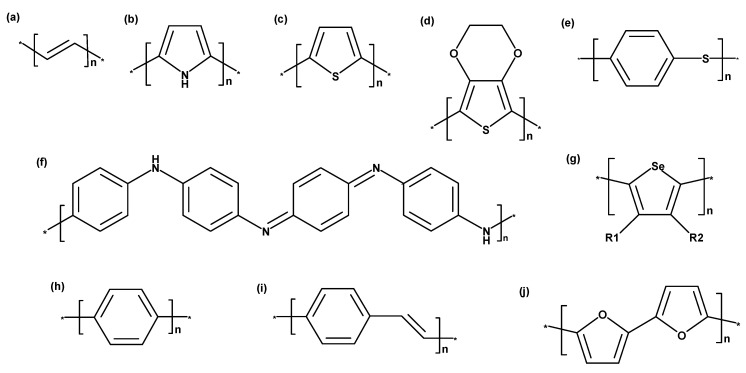
Structural formulae of intrinsically conducting polymers [1,2,3,4,5]: (**a**) Polyacetylene, (**b**) polypyrrole, (**c**) polythiophene, (**d**) poly(3,4-ethylenedioxythiophene), (**e**) polyphenylene sulfide, (**f**) polyaniline, (**g**) polyselenophene, (**h**) poly(para-phenylene), (**i**) poly(para-phenylene vinylene), and (**j**) polyfuran.

The structural formulae of intrinsically conducting polymers, such as PAc, PPy, PT, Polyphenylene sulfide (PPS), PAni, PPP, PPV, PFu, and PSe, are presented in Figure 3 [1,2].

### 3.1. Poly(Acetylene)

**Figure 4:**
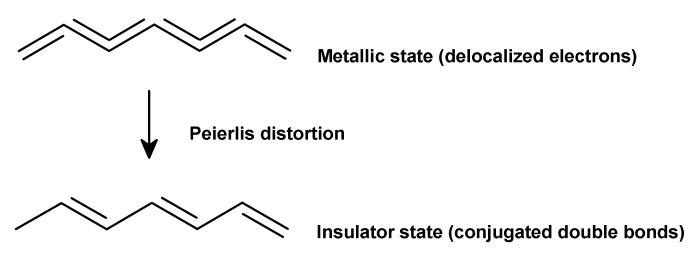
Effect of Peierls distortion on PAc in a neutral and undoped state [1,8].

PAc (Figure 3a), also called polyethyne, is a polymer of the monomer ethyne. There are three polyethyne isomers: trans-polyethyne, cis-polyethyne, and cis-cisoide polyethyne, with the latter having an unstable structure. In its ground state, PAc is an insulator due to the sufficiently long π-system that enables the localization of bonds (separation into double and single bonds). This process is termed Peierls distortion (Figure 4) [1,8] and ensures the separation of the fully occupied valence band (π-band) from the empty conduction band (π*-band) [8]. The resulting energy gap between bands can be avoided by means of thermal activation, which would however exceed the temperature resistance of ICP. Another option to avoid Peierls distortion involves doping in order to break double bonds, release electrons, and generate so-called solitons. The concept of solitons is based on a physical model describing charge transfer along a polymer chain. Solitons are capable of suppressing Peierls distortion locally. The more solitons there are, the higher the probability for interactions between them. Thus, the energy gap between bands as well as the distortion disappear. However, this concept can only be applied to the doping of PAc, since their energy level does not depend on the array of the polymer chain (degenerated state). Trans-PAc in a doped state possess a high electrical conductivity of 108 S/cm compared to other ICP (Figure 1) [8]. For other ICP, this solitons approach is not suitable, as it involves the separation of structures with different energy levels (non-degenerated state). They are referred to as polarons, which—in contrast to solitons—are not topologically stimulating and do not require a degenerated state in the polymer. Polarons are a double bond failure stabilizing the non-degenerated state; they consist of a neutral and a positively charged soliton [1,8].

Doped PAc was the first polymer for which a degree of electrical conductivity comparable with that of silver (being the most electrically conductive metal) could be observed (Figure 1). Although PAc offers the highest electrical conductivity, this conducting polymer has not yet been used commercially due to its low oxidation stability in the presence of oxygen and humidity. Furthermore, PAc shows a significant loss in conductivity after only 24 h. Hence, its insolubility in organic solvents, instability in air due to oxygen, and inconsistent conductivity make PAc difficult to process; it has therefore been replaced by semiconductor polymers, such as PPy, PAni, PPV, PF, and PT.

### 3.2. Polypyrrole

**Figure 5:**
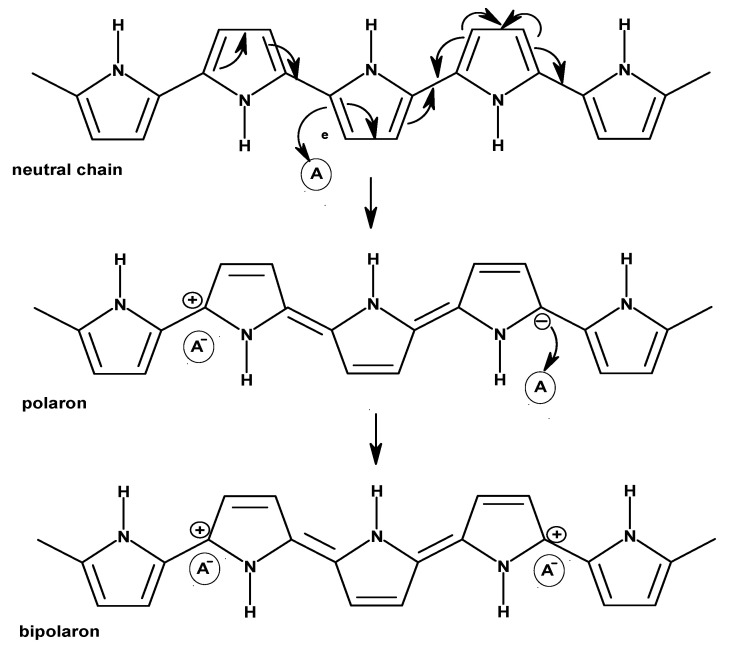
Polaron/bipolaron model of PPy [12].

PPy (Figure 3b) is a polymer consisting of 2,5-crosslinked pyrrole units. PPy is formed by the oxidative or electrochemical polymerization of pyrrol and belongs to the group of electrically conducting or conjugated polymers in the form of PHC. It was the first conducting PHC to be electrochemically synthesized, although it can also be directly produced in a doped form (similar as PT), and provides more stability in contact with air [1,8,9]. Further advantages of PPy compared with other ICP include its solubility in water, good capability of oxidation, and resistance of films to various environmental factors [10,11]. Charge transfer in PPy is performed by electrons and ions, whereby ions are transferred by migration and diffusion. The mobility and size of anions define the proportion of ionic conductivity in total conductivity. To achieve electronic conduction, a conjugated π-system along the polymer chain and “holes” generated during oxidation are required, which can result in a state of radical polarons or a state of non-radical bipolarons (Figure 5). Thus, electrons move along chains in the opposite direction to the positive charges (holes) of polarons and the state of bipolarons, analogous to a p-semiconductor. Moreover, electrons are also required to travel between chains. The system of conjugated double bonds combined with “doping” using a negatively charged counterion extends throughout the entire molecule, thus enabling conductivity. A stable conductivity of approx. 100 S/cm can be achieved for a prolonged period of time and a temperature of up to approx. 300 °C. With higher levels of doping, the band gap increases as well: in an undoped state, it measures 3.16 eV, whereas it amounts to 3.56 eV in a doped state. PPy possesses an electrical conductivity of 10^4^ S/cm in a fully doped state, which is a low value compared to, for example, PAc (Figure 1) [11,12].

PPy is often used for the manufacturing of electrically conducting textile materials, as its films are resistant to various environmental influences and it is soluble in water, can be easily oxidized, and offers conductivity at a stable level for a prolonged period of time.

### 3.3. Polythiophene

**Figure 6:**
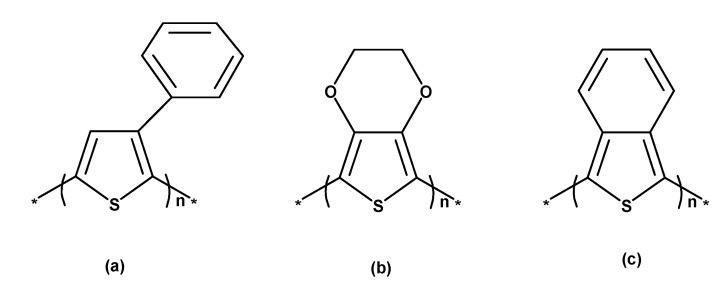
Examples of substituted PT: (**a**) Poly(3-phenylthiophene, (**b**) poly(3,4-ethylenedioxythiophene), and (**c**) poly(isothianaphtene) [5].

PT (Figure 3c) is a class of conjugated polymers typically employed for chemically and thermally stable materials. Disubstituted PT offers, in contrast to monosubstituted polymers, a greater potential for oxidation, an enhanced optical band gap, and low conductivity. The synthesis of PT is typically performed via electrochemical polymerization (preferred method), or alternatively, via Grignard coupling [8,9]. While PT in a neutral, undoped state has a conductivity of approx. 10^−8^ S/cm, this value can be increased to 1–100 S/cm by doping. In addition to alkyl chains, aryl groups (e.g., poly-(3-phenylthiophene), Figure 6a), ether and polyether chains, and redox groups are of great interest as potential substituents at the 3-position of PT. Substituents at the 3- and 4-position avoid the occurrence of 2,3-coupling during electropolymerization, which would decrease conductivity and cause over-oxidation of conducting polymers at higher potentials. Poly(3,4-ethylenedioxythiophene), short PEDOT, as presented in Figure 6b, belongs to this group; it has a high electrical conductivity (approx. 100 S/cm) and stability in a state of oxidation (Figure 1) [1,5]. Another representative of substituted PTs is poly(isothianaphthene), cf. Figure 6c [5].

Unsubstituted PT is insoluble and infusible; therefore, it is often manufactured as an alkyl-substituted derivate, e.g., poly-3-hexylthiophene (P3HT), by use of either electrochemical polymerization of 3-hexylthiophene or various, often organometallic, polymerization reactions [13]. Additionally, there is a large group of copolymers with substituted thiophenes, for example conducting polymers (e.g., pyrrole) [13] or graft copolymers with standard plastics (e.g., polystyrene) [14]. PT is, just like numerous polyaromatic compounds, insoluble in organic solvents due to its rigid backbone. The positive effect of a substitution in the 3- and/or 4-position of PT is limited to enhanced solubility [15]. Moreover, they are very cost-intensive (297 €/g) in comparison to other ICP, e.g., PAni (17 €/g), PPy (12.5 €/g), and PEDOT (3 €/g); in combination with their resistance to melting and insolubility, PT are unlikely candidates for the production of electrically conducting textile materials. In conclusion, only very few scientific publications have addressed PT so far.

### 3.4. Poly(3,4-Ethylenedioxythiophene)

In recent years, there has been a growing interest in PEDOT and its derivates compared to other ICP. PEDOT (Figure 3d) is among the most frequently used π-conjugated ICP [1]. Its favorable solution processability, electrical conductivity, and relatively low heat conductivity are advantages making this organic semiconductor an interesting material. Films made from this conducting polymer are optically transparent in their conducting state. However, their processability and application potential for specific devices are limited by their insolubility in water. Once the polymer is doped, it becomes relatively stable and achieves a satisfying level of electrical conductivity (100 S/cm). PEDOT was combined with water-soluble polymers, such as poly(styrene sulphonic acid) (PSS) to improve its solubility in water. PEDOT:PSS offers both high conductivity and favorable transparency in visible areas in addition to excellent thermal and hydrolytic stability; hence, it can be easily processed in aqueous dispersions using spin coating. In an industrial context, PEDOT:PSS is synthesized from an ethylenedioxythiophene (EDOT) monomer and PSS by use of sodium persulfate acting as oxidizing agent. The result of this process is PEDOT in its highly conductive, cationic form. PSS with its significantly higher molecular weight functions as counterion, thus keeping PEDOT chain segments dispersed in an aqueous medium. In general, PEDOT:PSS hydrogel particles offer excellent processing properties for the production of thin, transparent, conducting films [16]. As a result of their solution processability, stable electrical conductivity, transparency in the conductive state, and low price (3 €/g) in comparison with other ICP, PEDOT is often selected for producing electrically conducting textile materials [17,18].

### 3.5. Polyaniline

**Figure 7:**
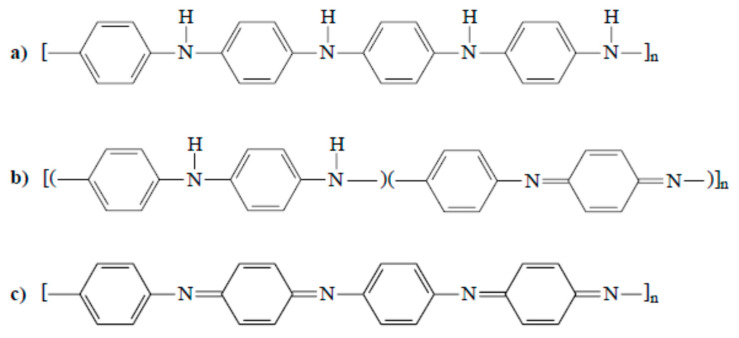
Different oxidation states of PAni: (**a**) fully reduced LeucoEmeraldine Base (PAni-LEB), (**b**) half-oxidized Emeraldine Base (PAni-EB), and (**c**) completely oxidized PerNigraniline Base (PAni-PNB) [20].

PAni (Figure 3f) is a radical-cation salt of a conjugated polymer with oxidatively coupled aniline units and an acid, which is self-conducting in salt form without further additives. For example, PAni can be dissolved in N-methyl-2-pyrrolidone (NMP). However, doped types of polymers are in some cases not suitable for thermal processing. In terms of PAni synthesis, different influencing factors come into play, such as the selected monomers, solvent, conducting salt, and oxidation conditions, thus affecting the chemical composition and morphology. Additionally, the color and characteristics of PAni vary according to their oxidation state; three important states can be identified as follows [6,19,20]:

- Light yellow/colorless oxidation state: reduced form of the stable “green polymer”, quickly oxidizes when exposed to air, non-conducting (Figure 7a).
- Blue oxidation state: stable form of the polymer, neutral non-conducting form; can be generated from the “green” type of PAni by neutralization using bases (Figure 7b).
- Green oxidation state: most stable form of the polymer. This form of oxidation is the most commonly used oxidation state of PAni; called conducting or organic metal (Figure 7c).

One of the most commonly investigated conjugated ICP is PAni. At room temperature and even at higher temperatures, PAni offers a comparably high conductivity and stability. This advantage, combined with its availability, non-toxicity, and processability by coating, printing, or spinning, makes PAni suitable for the manufacturing of electrically conducting materials on an industrial scale for various potential applications. However, material costs in the case of PAni (17 €/g) are high, especially compared to other ICP, such as PPy (12.5 €/g) and PEDOT (3 €/g) [18].

### 3.6. Poly(Para-Phenylene)

Unsubstituted PPP (Figure 3h) is produced by a precursor route consisting of cyclohexa-3,5-diene-dicarboxylic acid esters. In accordance with most conducting polymers, PPP is insoluble and infusible. The soluble prepolymers generated during production merge into PPP as a result of heating and forming processes. The polymer itself is more resistant to oxidation than PAc and can therefore only be doped by means of arsenic pentafluoride (AsF5), thus achieving extremely high conductivities of up to 10^4^ S/cm [1]. In a single-step reaction, for example, para-phenylene-trimers can be transformed into a metallic, shiny, blue, conducting polymer using AsF5 steam. PPP is very temperature-resistant and can withstand air temperatures of up to 500 °C without destruction. In contrast to PAc, PT, and PEDOT, only very few researchers have so far investigated PPP due to its insolubility and infusibility, making it unsuitable for the manufacturing of electrically conducting textile materials.

### 3.7. Poly(Para-Phenylene Vinylene)

PPV (Figure 3i) is a bright yellow, fluorescent, conducting polymer with an acceptable emission maxima. As it is insoluble and infusible, its production requires a soluble precursor polymer. It can then be processed into films, and subsequently, into highly molecular PPV due to thermal treatment [1,2,3,4,5]. PPV is a diamagnetic material with a very low potential for intrinsic electrical conductivity in the range of 10^−13^ S/cm. This value can be enhanced by adding iodine, ferric chloride, alkali metals, acids, or other doping agents. Nevertheless, the stability of the resulting doped materials remains relatively low. In general, unaligned and unsubsituted PPV are characterized by a moderate conductivity level, which can be increased by doping to up to <10^−3^ S/cm when using iodine, and 100 S/cm in the case of sulfuric acid [1,2,3,4,5]. Alkoxy-substituted PPV are typically easier to oxidize than PPV, thus offering more favorable conduction capabilities. Longer side chains decrease conductivity and impede the formation of disulfide interchain bonds due to the hopping of charge carriers. PPV, similar to PAc, is difficult to process for electrically conducting textile materials, since it is unstable in a doped state, insoluble, and infusible.

### 3.8. Polyfuran

PFu (Figure 3j) is a polymer consisting of multiple furanylene rings. These types of material are of great interest for their potential in molecular electronics, although they have been significantly less researched than PT, PPP, and PPV, and are hardly ever used for the manufacturing of electrically conducting textile materials. PFu can be prepared using electrochemical approaches, or alternatively, acid catalysts [21].

## 4. Methods and Procedures

Although ICP are of great interest to the scientific community, their processability still poses a great challenge for researchers, since they are typically infusible, moisture-sensitive, and unstable at high processing temperatures; moreover, they are insoluble in most standard solvents and unstable when exposed to air. This chapter will introduce methods and procedures for the realization of electrically conducting textile products made from ICP.

### 4.1. Printing Technologies

**Figure 8:**
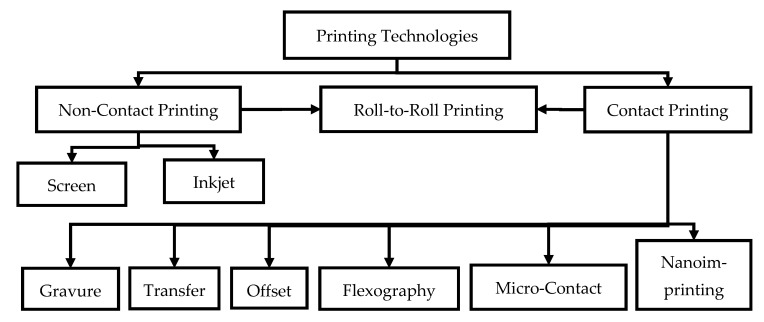
The classification of common printing technologies for ICP.

Throughout the past years, several different printing technologies (Figure 8) have been developed in order to apply ICP onto various textile substrates. There are contact and non-contact printing techniques, cf. Figure 8. For the non-contact printing techniques, the transfer of the intrinsic conductive printing paste takes place in a printing block or plate (e.g., stencil for screen printing, jet for ink jet printing) onto the textile. Here, the necessary pressure of contact is a result of the mechanical pressure of the squeegee. Contact-based printing technologies include gravure printing, micro contact printing, nanoimprinting, and transfer printing. Non-contact printing techniques comprise screen printing and inkjet printing. For the contact printing, the application is realized by the direct contact of a transfer subcarrier (e.g., rubber blanket for offset printing, foil for flexography, rubber cylinder for gravure printing) with a gentle pressure onto the textile. The next sections will give an overview on various printable ICP and different substrate materials in addition to describing the advantages and disadvantages of all relevant printing technologies. The primary benefits of printing compared to coating are low chemicals consumption, accurate printing on a substrate by means of printing paste, less residual solution, and the cost-effective manufacturing of large-scale conducting textile materials. In contrast, a significant disadvantage of current technologies is that the conducting layer is connected only on the fiber surface.

Different printing technologies require different parameters, e.g., in terms of viscosity and surface tension of the printing paste, conductivity and compatibility of the solvent, and bond strength between printing paste and underlying substrate. To simplify the processing process, ensure the precise deposition of ICP, reduce the demand for ICP, increase the throughput volume of the paste as well as production speed, and establish a cost-efficient technique suitable for mass production, numerous teams of researchers have investigated various printing options.

To achieve a satisfactory quality and electrical conductivity of the intrinsic conductive polymer film, different additives are used, such as ammonium peroxydisulfate (APS), dodecyl benzene sulfonic acid (DBSA), or PPS, in the ICP solutions such as PAc, PAni, PPy, or PEDOT [22]. Next to the former explained additives, organic semiconductors, such as poly(3-hexylthiophene) (P3HT), poly(triarylamine), poly(3,3-didodecyl quaterthiophene), poly(2,5)-(3-tetradecyllthiophene-2-yl), and thieno(3,2-thiophene) are inexpensive, solution processable, and having acceptable charge transport compared to ICP. Therefore, the organic semiconductors were mixed with intrinsic conductive polymers, to achieve an inexpensive, homogeneous, and more electrically conductive material. To improve the solubility of the ICP, organic dielectric materials were used, such as poly(4-vinylphenol) (PVP), poly(methyl methacrylate) (PMMA), PET, polyvinylidene fluoride (PVF), polyimide (PI), polyvinyl alcohol (PVA), and polystyrene (PS), for printing technologies [22,23,24,25,26,27,28,29,30,31,32,33,34,35,36,37,38,39]. Suitable thickeners and binders are, for example, carrageenan’s, thickeners based on polyurethane, polysaccharides, polyacrylates, polyvinylpyrrolidone, polyethylene oxides, agar agar, tragant, gum arabic, alginates, pectins, guar flour, carob kernel flour, starch, dextrins, gelatine, casein, carboxymethylcellulose and other cellulose ethers, hydroxyethylcellulose, hydroxypropylcellulose, polyurethanes, polyvinyl acetates, polystyrene, polycarbonate, polyester (PES), polyvinyl alcohol, and PA, in order to achieve suitable viscosity and surface tension of the printing paste for the various printing processes [25]. It is also possible to add fillers to the printing pastes in order to achieve the desired rheology. Suitable fillers are metal oxides, such as titanium dioxide, zinc oxide, and aluminium oxide; electrically conductive metal oxides, such as indium-tin oxide and antimony-tin oxide; metals, such as silver, copper, gold, palladium, and platinum; silicon dioxide, silicates, silicas, polysilicic acids, zeolites, alkaline earth metal carbonates, such as calcium carbonate, phyllosilicates, and clay minerals, such as montmorillonites or bentonites [25]. Furthermore, it is possible to add crosslinking agents, such as epoxysilanes (for example 3-glycidoxypropyltrimethoxysilane), silane hydrolysates (for example hydrolysates of tetraethoxysilane), or di- or oligoisocyanates to the printing pastes, to bond strength between printing paste and underlying substrate [25]. Small amounts of glycerol, sorbitol, dimethyl sulfoxide (DMSO), N-Methyl-2-pyrrolidone (NMP), ethyleneglycol (EG), etc. were used as doping agents [22,23,24,25,26,27,28,29,30,31,32,33,34,35,36,37,38,39]. Not only textile surfaces, but also glass, metal, and plastic sheets, were tested as potential substrates. Glass is sensitive to the transverse force, so it breaks off quickly. In contrast, metals can withstand very high temperatures, while still being an unfavorable option for the application of ICP in flexible electronics due to the roughness of their surface and high costs involved. The main obstacle for the application of plastics, such as PET, glass temperature: (T_g_) = 70 °C), polystyrene (PS, T_g_ = 100 °C), polycarbonate (PC, T_g_ = 150 °C), polyacrylate (PAC, T_g_ = 210 °C), or polyimide (PI, T_g_ = 270 °C), as substrates have significantly lower glass transition temperature (T_g_) compared to metals. Textile materials offer great flexibility and elongation in contrast to metals. Optimal substrates should provide dimensional and thermal stability, a low coefficient of thermal expansion, excellent resistance to solvents, and good barrier properties in terms of humidity and gas. So far, mainly non-contact printing technologies, i.e., screen and inkjet printing, have been used to apply thin intrinsically conducting films onto various textile surfaces (Table 2).

#### 4.1.1. Screen Printing

Screen printing is an environmentally friendly, highly efficient, and yet relatively simple method for the production of complex electronic circuits and links. This technology is based on simple equipment, i.e., a screen, scraper, printing bed, template, printing paste, and substrate. As part of the screen printing process, a liquid printing paste is pressed through a screen (template) using a rubber scraper, thus creating the desired pattern on the substrate surface. The screen printing process is widely used among textile manufacturers due to its robustness, simplicity, large throughput volume for printing paste, and low costs involved in mass production. For the screen printing, a higher viscosity (1–20 Pa*S) of the printing paste and a thicker print layer (0.01–500 μm) are needed compared to inkjet printing, and it exhibits a slower printing speed (90–5400 m/h) [40].

Seiichi Takamatsu et al. [23] used polyimide (PI) film for the printing template due to its hydrophobic and temperature-resistant properties. To create a low-resistance layer, an additional layer of polydimethylsiloxane (PDMS) including an ionic liquid gel was applied onto the PI printing template. The resulting PI-PDMS template was placed on the PES fabric, and the aqueous conducting PEDOT-PSS solution was applied via screen printing (Yuzen method). Once dried, the PDMS layer remained on the fiber, whereas the PI layer was removed. Wiesława Urbaniak-Domagała et al. printed PPy/graphene/carbon nanotubes onto knitted fabrics made from polyurethane (PU) and polyamide 6 (PA6) [24].

The US patent US 6,358,437 Bl describes a screen printing method for manufacturing a conducting PES fabric based on PEDOT:PSS printing paste [25]. Victor et al. [26] applied PEDOT or PAni by means of the adhesive agent indium tin oxide onto PES or PA fabrics, whereas V. Rubežienė et al. printed PEDOT:PSS onto CO and PES fabric by screen printing [27].

Moreover, a specific pattern was created on textile gloves made of PET, elastane, and piezoelectric poly(vinylidene fluoride) (PVDF) yarn by use of PEDOT:PSS printing paste and screen printing. The PES or PA surface was prepared with plasma or wet-chemical treatments. In this context, Performax XPE1210 was used as binding agent and ethylene glycol as doping agent [28].

PEDOT is the most commonly applied ICP for screen printing, whereas PET or PA were typically selected as textile substrate. The printing quality and properties depend on various factors, such as the viscosity and surface tension of the printing paste, surface energy and strength of the substrate, printing speed, angle and geometry of the scraper, the gap between screen and substrate, mesh size, and material selection (CO, PET, PA, PU, and PVDF). The solvent ensures the required viscosity for the printing procedure and volatility for thermal curing, while the binding agent enhances the mechanical strength of the printing paste and its bonding strength on the substrate. To achieve proper bonding on textile surfaces, the fiber surface is prepared by chemical or thermal pretreatment. Subsequent to the printing process, the recently deposited printing paste is cured by use of heat or UV radiation.

#### 4.1.2. Inkjet Printing

The very commonly used inkjet printing technology is based on either thermal or piezoelectric drop-on-demand printing heads. Thermal printer heads are equipped with a resistive element in every nozzle. Upon heating of the resistor, the ink achieves its melting temperature, thus forming a vapor bubble. Typically, bubble formation occurs within 2 microsiemens at a temperature of approx. 300 °C. As the bubble expands, the pressure within the nozzle increases, thus propelling a droplet of ink out of the nozzle. Subsequently, pressure decreases and the nozzle is refilled with ink. The second type of printing head involves piezoelectric crystalline material in an ink-filled chamber behind each nozzle. When voltage is applied, the piezoelectric material expands, generating pressure in the fluid and forcing an ink droplet from the nozzle. Piezoelectric inkjet technology is often preferred over thermal printing, as it does not require high temperatures of 200–300 °C that potentially cause thermal degradation of ink, particularly when containing organic or biological materials. However, the handling of thermal inkjet printing mechanisms is easier than piezoelectric mechanisms; it allows the use of environmentally friendly solvents, and fewer ink additives are required. Moreover, less ink viscosity and surface tension are needed for thermal inkjet printing; hence, a viscosity of 1–1.5 cPs is sufficient for thermal inkjet printing, while a viscosity of 5–10 cPs and a surface tension of 30 Dyn cm^−1^ are the minimum values for piezoelectric inkjet printing [29,30]. In general, inkjet printing offers several advantages over screen printing, i.e., a thinner print layer (0.01–0.5 µm), increased printing speed (0.02–5 m/min), lower viscosity (0.001–0.1 Pa·S), and lower surface tension (15–25 mN/m) of ink. Hence, even an extremely small amount of printing paste can be deposited in a fast one-step process, thus enabling very precise patterns with high resolution and enhanced reproducibility. Potential disadvantages of this method include nozzle clogging, long solvent vaporization periods, bubble formation in the printing head, and fuzzy contours of the printed pattern on the substrate. In addition, the required high temperatures may damage the substrate. In conclusion, these drawbacks make inkjet printing an unsuitable option for industrial production.

Nevertheless, inkjet printing has great potential for pattern printing using conjugated polymers in high resolutions. The technology principle is based on the controlled deposition of small amounts of ink through an individual nozzle, or a combination of nozzles. Thus, extremely small droplets can be placed onto a substrate with high precision and reproducibility. A suspension containing PEDOT:PSS was selected for printing onto PA and mercerized CO by Sawhney et al. [31,32] based on a two-step inkjet printing process. The same team of researchers also employed inkjet technology to apply silver nitrate lines onto nylon using currentless metallization with ammonia as complexing agent and glucose as reducing agent. Between those silver lines, PEDOT:PSS was printed. PSS acts as doping and dispersing agent, while simultaneously balancing charges. After printing, the conductivity of PEDOT was at approx. 25 S/cm [33,34]. Moreover, innovative benzidine-free PAni inks suitable for electrically conductive inkjet printers were developed and tested [35]; a pattern was applied onto CO and PES fabric by means of piezoelectric single-use inkjet printing technology. In this context, the influence of printing method and droplet size on the quality of the print was discussed [36]. Various techniques, including plasma treatment [37] or adjusting adhesion/cohesion properties of printing pastes, were investigated to achieve optimally printed patterns on flexible substrates. In many cases, wetting agents, pigments, and polymeric compounds were added to improve the resolution and quality of the resulting print film. For example, the tenside Tween 80 was added to an aqueous PEDOT:PSS dispersion to achieve a printing paste with the desired surface tension for thermal printing [38]. To generate a paste with a suitable viscosity level, Ngamma et al. evaluated options made of a PAni nanoparticle dispersion, ammonium persulfate (APS), dodecylbenzenesulphonic acid (DBSA), and aniline; they were then used for piezoelectric inkjet printing. It was observed that a high concentration of DBSA tenside led to an increase in the specific resistance of the PAni dispersion, and a low amount of APS generated high conductivities [22,36]. In conclusion: although inkjet printing offers numerous advantages, it is difficult to create an ink that can be used for ICP and textile fiber surfaces.

#### 4.1.3. Gravure Printing

Gravure printing is among the oldest and simplest printing techniques used in an industrial context. For this technique, patterns are engraved on the impression cylinder, and the printing paste is transferred from its container to the engraved metal cylinder. Subsequently, excess printing paste is removed with a scraper, leaving only the depressions filled with paste. Due to a large contact pressure and adhesive forces between paste and textile, the paste is transferred. A printing paste containing graphene/PEDOT:PSS/ethyl cellulose was applied onto PET using a roll-to-roll gravure printing technique [39]. Future research projects are anticipated to focus on the modification of ICP as ink and the interactions between ink and substrate for roll-to-roll gravure printing methods. Currently, there are hardly any research publications on the application of ICP on textile material surfaces using gravure printing. Numerous influencing parameters, such as the surface porosity, penetration depth of polymers, surface roughness, and evenness of the resulting film on the textile surface, should be addressed in the future.

#### 4.1.4. Transfer Printing

**Figure 9:**
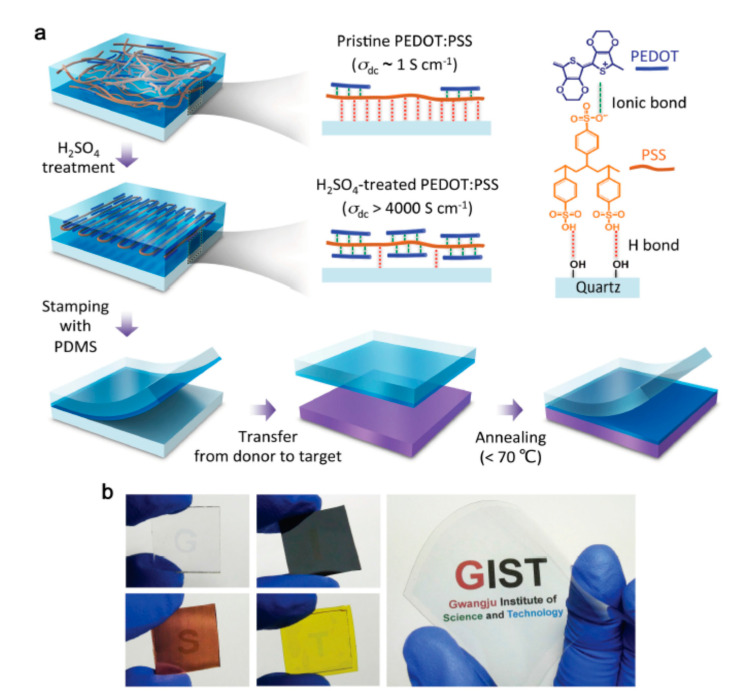
Transfer process for highly conductive PEDOT:PSS films. (**a**) Schematic illustration of the procedure for the transfer printing of H_2_SO_4_ -treated PEDOT:PSS film from a donor quartz substrate to a target substrate. The mechanism of transfer printing is based on the chemically controlled adhesion of the PEDOT:PSS to the substrate via H_2_SO_4_ treatment. (**b**) Photographic images of H_2_SO_4_-treated PEDOT:PSS films transferred onto various substrates, including a glass slide, a silicon wafer, Cu foil, Kapton tape, and large-area PET foil [41].

Transfer printing belongs to the class of contact-based techniques and can be described as a procedure where—in contrast to direct printing—the mirrored motif is first printed onto a so-called transfer paper or foil using special binding agents or pastes. The motif on the prepared transfer foil is then printed onto textiles via heat or pressure. Kim et al. first produced a foil made of PEDOT:PSS, which was then treated with sulfuric acid in order to dope PEDOT (Figure 9). The resulting doped PEDOT:PSS foil was printed onto a transfer foil consisting of polydimethylsiloxane (PDMS). In a final process step, the preprinted PEDOT:PSS/PDMS foil was transferred to a PET foil at 70 °C. The highly transparent PET/PEDOT:PSS foil can be used as OLED electrode due to its high conductivity (>4000 S/cm) and transparency (90%), excellent flexibility (bending radius <400 µm), and great quality [41]. Nevertheless, transfer printing is a very time-consuming procedure, as a new transfer foil must be prepared for each paste. In general, this process involves numerous work steps leading to relatively high costs. Therefore, transfer printing is mainly used in the high-quality sector for customized products.

#### 4.1.5. Offset Printing

**Figure 10:**
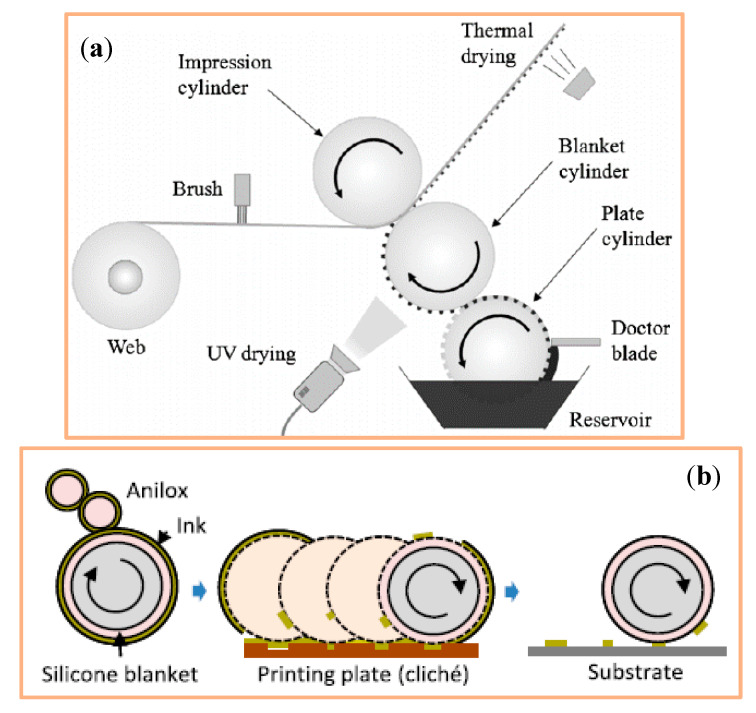
(**a**) Schematic of a typical micro-gravure-offset printing process [42]. (**b**) Process schematic of the modified offset roll printing [43].

Offset printing is an indirect flat printing technique where printing plate and substrate do not come into contact with one another. First, the printing paste is transferred to a blanket cylinder, then to the printing surface (Figure 10) [42,43]. Therefore, the printing plate is preserved and can be reused for numerous types of blanket, such as Teflon, polymethylmethacrylate, and of film substrates polyester, polyvinyl chloride, polyamide or polyimide [42,43]. So far, no research works have been published on this specific textile related topic. Hence, an important future task for researchers in this field is the development of suitable ICP pastes for this technique.

#### 4.1.6. Flexography

**Figure 11:**
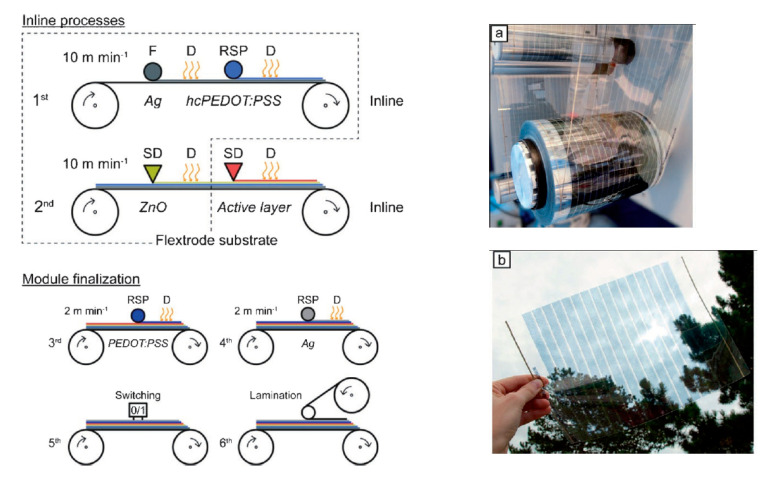
Left: Fast inline roll-to-roll printing and coating on PET for the manufacture of indium-tin-oxide-free (ITO-free) polymer solar cells comprising a six-layer stack: silver-grid/PEDOT:PSS/ZnO/P3HT:PCBM/PEDOT:PSS/silver-grid. The illustrated processes are flexo-printing (F), rotary-screen printing (RSP), slot-die coating (SD), and drying (D). Right: (**a**) Hundreds of meters of flextrode substrate (PET) ready for further R2R processing. (**b**) Flextrode substrate with 16 individual electrode stripes [44].

The initial process step of flexography involves the transfer of printing paste onto the so-called anilox roll and the removal of excess paste using a scraper. Next, the printing paste is moved from the anilox roller to the flexo plate that holds the desired image, which is then transferred to the textile. For example, six layers (silver-grid/PEDOT:PSS/ZnO/P3HT:PCBM/PEDOT:PSS/silver-grid) were printed onto PET foil by means of flexography (Figure 11) [44]. However, inks and printing pastes must be further modified to make them suitable for commercial use; for example, the adhesion between substrate and flexo plate must be optimized [44]. In comparison, the chemical and physical characteristics of printing paste and substrate are of greater importance for flexography than for gravure printing. Roll-to-roll flexo printing is relatively cost-effective and enables the realization of a large variety of designs. This method is suitable for water- and oil-based inks. However, it is rarely used for the manufacturing of electrically conducting textile materials made of ICP. Very few scientific publications have addressed this topic so far. Due to its acidic effect on rubber and flexible foils, high evaporation rate, and low viscosity, it poses considerable technical challenges on an industrial scale.

#### 4.1.7. Nanoimprinting

**Figure 12:**
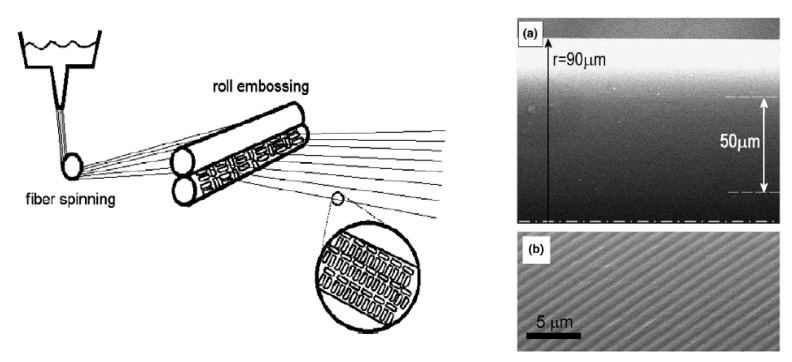
Left: Fabrication setup for lateral surface structuring by roll embossing. The fibers are either still soft from the spinning process or softened on the surface by heating them locally. Right: SEM micrographs of a roll embossed PES fiber. (**a**) The patterned area with the inclined molded grating is indicated by the arrows on the right side. (**b**) Magnification showing the replicated 1 µm sine grating [45].

Roll-to-roll nanoimprinting is a promising option for the integration of nanoscale electronics and optoelectronics based on ICP. It is a high-speed method for the generation of layers with thicknesses in the sub-micrometer range on textile surfaces. Moreover, nanoimprinting is a particularly interesting approach if combined with other roll-to-roll techniques. In the industry, roll-to-roll nanoimprinting processes (Figure 12) are employed for thermoplastic polymers or films, and for the structuring of PES textile fibers [45]. However, scientific publications on this topic are scarce.

In conclusion, it can be said that presently, there is no ideal printing technology for the application of ICP on textile substrates that could enable the mass production of patterns on the nanometer scale at a high production rate and printing speed. Up to now, PEDOT has been the most widely used ICP for the printing technologies introduced in this chapter. PET and PA were typically employed as textile substrates. The most frequently discussed and used printing technology has been screen printing, although rarely occurring in combination with other printing methods. The future development of these technologies will aim for a one-step process with a large throughput volume, high production speeds for small pattern sizes (including nano-sized structures), high precision, and proper interaction between ICP and fiber surfaces.

### 4.2. Fiber Spinning

**Figure 13:**
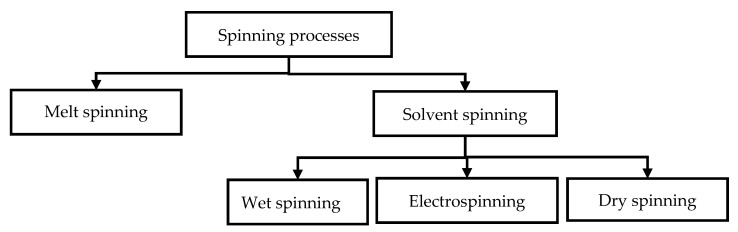
The classification of fiber spinning technologies for intrinsically conducting polymers.

Electrically conducting textile materials based on ICPs can also be produced by means of spinning technology (Figure 13); additionally, this technology allows the production of electrically conducting staple or filament yarns based on ICPs. Thus, electrically conducting fibers are spun from an ICPs solution based on electro, wet, or dry spinning techniques. In the case of electro spinning, a liquid containing ICPs substances is spun by exploiting a strong electrical field. A return electrode is located at a specific distance (several centimeters) and acts as either counter pole or—in most cases—grounded electrode. Moreover, wet and melt spinning are also suitable options for the manufacturing of conducting fibers. During wet spinning, a textile material is pressed through a spinneret and subsequently treated with solvent (mixtures) in precipitation or coagulation baths. Hence, the polymer precipitates, and after several other processing steps (washing, drawing, drying, coating), individual or multiple filaments can be wound as yarns. Dry spinning is a type of solvent spinning where a polymer/solvent solution is extruded in a heated spinning chamber, into which hot gas steam is blown; thus, after passing the spinneret, the yarn is free from organic solvents and has transformed from gel into a solid state. Subsequently, yarns are drawn and wound. For melt spinning, pretreated granules are melted using extruders. Polymer melts are heated to or above their melting point (depending on the polymer) and pressed through spinnerets, whereas the generated melt jets are solidified by the application of various post-treatment measures, thus forming thin fibers.

#### 4.2.1. Electrospinning

**Figure 14:**
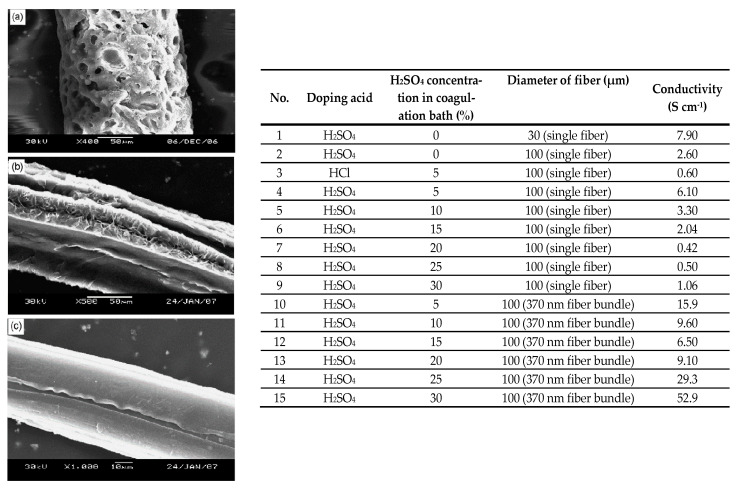
Left: SEM images of H_2_SO_4_-doped PAni fifibers obtained from different H_2_SO_4_ concentration contained in coagulation bath: (**a**) 15%, (**b**) 5%, and (**c**) 0%. Right: Table summarizes the conductivity of PAni sub-micron fibers from electrospinning [47].

Electrospinning (Figure 13) is the most widely used technology for the production of electrically conducting nanofibers, as it is easy to handle, very versatile, and enables the manufacturing of extremely thin fibers and nanofibers (100 nm–50 µm) based on various ICPs (Table 3). So far, PAni has been the most extensively researched and investigated ICP for spinning. Its beneficial processing properties, stability, and adjustable electro-optical characteristics make this polymer particulary suited to electrospinning. However, its insolubility creates a considerable challenge for PAni electrospinning [46]. Yu et al. manufactured PAni nanofibers based on electrospinning, while adding sulfuric (Figure 14, left) and hydrochloric acid as doping agent and solvent, respectively. Moreover, an additional coagulation bath containing diluted sulfuric acid solution was used. Electrical conductivity was modified in the range of 7.90–52.90 S/cm by adjusting the fiber diameter. For example, a conductivity of 52.9 S/cm was achieved with a fiber diameter of 370 nm (Figure 14, right) [47].

**Figure 15:**
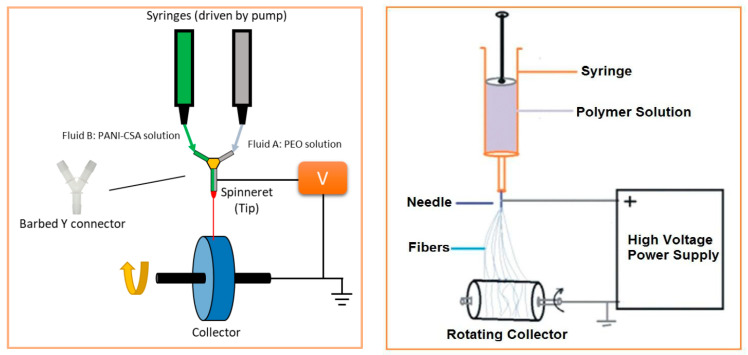
(**Left**) Schematic illusion of side-by-side electrospinning. PAni-CSA: Camphoric acid doped Polyaniline; PEO: polyethylene oxide [49]. (**Right**) Schematic diagram of electrospinning for nanofibers fabrication [53].

Another challenging task was to spin fibers with the desired level of electrical conductivity. In many cases, the solution approach to this issue involved the mixing of PAni and other spinnable polymers. Rutledge et al. for example used an aqueous (+) Camphor-10 sulphonic acid solution (HCSA) as doping agent and solvent. HCSA-doped PAni was mixed with PMMA or PEO, and subsequently, fibers were produced via electrospinning. Conductivities of up to 50 ± 30 S/cm were achieved [48]. Liu et al. introduced the side-by-side electrospinning of PAni fibers (Figure 15, left). These fibers were manufactured from two solutions, one consisting of Camphor acid (CSA) doped PAni and the other one of PAni mixed with PEO. However, the resulting side-by-side PAni–PEO fiber exhibited undesirable mechanical properties, since the two components are immiscible, thus leading to a lack in chemical bonds and interface adhesion [49].

Other types of electrically conducting fibers were generated by electrospinning using a mixture of PAni, Camphor sulphoric acid, and PEO [50]. Some researchers added extrinsically conducting materials, e.g., carbon nanotubes (CNT), to the PAni spinning solution to enhance the stability of the resulting fibers [51]. Furthermore, conducting nanofibers were also manufactured using electrospinning with a mixture of multi-walled carbon nanotubes (mwCNTs), PAni, and PEO [52]. Shrikant et al. presented nanofibers made of a PAni/titanium dioxide (TiO_2_) nanocomposite that were also manufactured using electrospinning technology (Figure 15, right). These nanofibers exhibited a high sensitivity of 1000 ppm to CO_2_ gas at a low operating temperature of approx. 48 °C [53]. Numerous scientific publications describe PAni/CNT compounds [54] and PAni/silica fibers [55], where ICP were mixed with extrinsically conducting fillers to increase their electrical conductivity.

Furthermore, the manufacturing of structures with a conducting polymer covered by an insulating polymer offers great potential; this approach is anticipated to generate the functions of a conducting insulator-nanostructure and improve the processability and mechanical properties of conducting polymers by electrospinning. Wei et al. [56] produced electro-spun core/sheath nanofibers based on PAni blends by mixing various polymers (carriers), such as PSS, PC, PMMA, and PEO, in the spinning mass.

Depending on the polymer type of the carrier, the electrical conductivity of spun nanofibers increased from 5.5 × 10^−14^ S/cm to 2.4 × 10^−13^ S/cm. Ultra-thin PEO fibers were spun from a combination of PAni and cadmium sulfide (CdS) nanoparticles. It was observed that the introduction of PAni led to a significant increase in photoluminescence. This material type can be applied for the production of electroluminescent and nanooptoelectronic devices [57]. Moreover, Zhao et al. [58] discussed the manufacturing of PEDOT/PSS/PEO nanofibers using electrospinning, whereby highly molecular PEO were processed as carriers. PEDOT is a linear conjugated polymer with a low band width of 1.5–1.6 eV and attracted attention from the scientific community due to its excellent electrochemical stability and transparency in a doped state. Single electro-spun fibers reached electrical conductivities of up to 35.5 S/cm [58].

Liu et al. developed a nanofiber made from PEDOT/PSS/polyvinyl alcohol with dimethyl sulfoxide encapsulated on Kapton film using electrospinning technology. The conductivity of electro-spun PEDOT/PSS–PVA Kapton film was in the range of 4.8 × 10^−8^–1.7 × 10^−5^ S/cm [59]. In addition, a nonwoven material was produced from a solution containing PVDF/PPy/CuCl_2_·2H_2_O and *N*,*N*-dimethylacetamide (DMAc) by electrospinning. To increase its electrical conductivity, the nonwoven material surface was coated with mwCNT; the resulting electrical conductivity of the PPy-mwCNT compound was 10^−1^ S/cm [60].

Recently, the integration of ionic liquids into polymers has gained interest in order to enhance conductivity and promote electron transfer. Savest et al. produced conducting mats based on PAni and blends of ionic liquids using electrospinning [61]. Lee et al. generated a nanofiber using P3HT and poly(ε-caprolactone) [62]. Electrospinning technologies also enabled the manufacturing of poly(3-hexylthiophene) (P3HT) nanofibers by adding iodine as doping agent. As a result, the electrical conductivity of individual P3HT nanofibers amounted to 122 ± 9 S/cm. The tensile strength of nanofibers was measured to be 4.6 ± 0.2 MPa, whereas their elongation at break was at 24.6 ± 2.2% [63]. A nanofiber consisting of PPy and poly(lactic-co-glycolic acid) (PLGA) was prepared by electrospinning and introduced by Y. Lee et al.; this nanofiber type is suitable for use in neuronal tissue scaffolds [64].

In conclusion, it can be stated that it is possible to produce high-quality electrically conducting nanofibers by means of electrospinning. However, a successful manufacturing process depends on a large number of factors, including solution properties (viscosity, conductivity, surface tension), process parameters (field strength, flow rate, spinning distance, tube, solution temperature), and environmental parameters (air humidity, ambient temperature). The most frequently used ICP in electrospinning were PAni, PEDOT, PPy, and P3HT. PEO, PMMA, PC, PS, or PLGA were often employed as organic dielectric materials, and hydrochloric, sulfuric, or (+)Camphor-10 sulfonic acid, iodine, dimethyl sulfoxide, *N*,*N*-dimethylacetamide, N-methylpyrrolidone, or ethylene glycol were used as doping agents. Moreover, TiO_2_ was applied for protection against oxidation. To increase electrical conductivity to the desired level, extrinsically conducting fillers, e.g., swCNT or mwCNT, were added to the spinning material. Not only individual, but also bicomponent nanofibers were generated via electrospinning. Although these fibers are highly conductive, they provide low ductility, little to no elasticity, and an insufficient amount of tensile strength.

#### 4.2.2. Wet Spinning

Wet spinning technologies not only enable the production of electrically conducting staple fibers, but also of filament yarns made from ICPs. During wet spinning, organic as well as inorganic solvents can be used. In addition to the advantage of producing both staple and filament yarns, this spinning method requires low processing temperatures. Conducting microfibers were manufactured using PSS-doped PEDOT with a diameter ranging between 4.6–16 µm via wet spinning. Acetone was selected for the coagulation bath. The microfibers obtained were characterized by oriented polymer chains, low crystallinity, and an electrical conductivity of 10^−1^ S/cm regardless of the diameter. The corresponding Young’s modulus, tensile strength, and elongation at break were 1.1 × 0.3 GPa, 17.2 × 5.1 MPa, and 4.3 × 2.3%, respectively [65]. Six years later, Okuzaki et al. manufactured a highly conducting microfiber based on PEDOT doped with poly(4-styrenesulfonate) using wet spinning; they achieved enhanced electrical conductivities by a post-treatment that involved the immersion of fibers into an ethylene glycol (EG) bath and subsequent tempering for 30 min at 160 °C. Okuzaki et al. revealed that the PEDOT:PSS became significantly softer during EG post-treatment, thus improving the mechanical properties of the resulting microfiber. The Young’s modulus increased from 3.2 to 4.0 GPa and the tensile strength from 94 to 130 MPa. Moreover, the two-step process led to an increase in conductivity by a factor of 6 and up to 17 (from 195 S/cm to 467 S/cm) [66].

**Figure 16:**
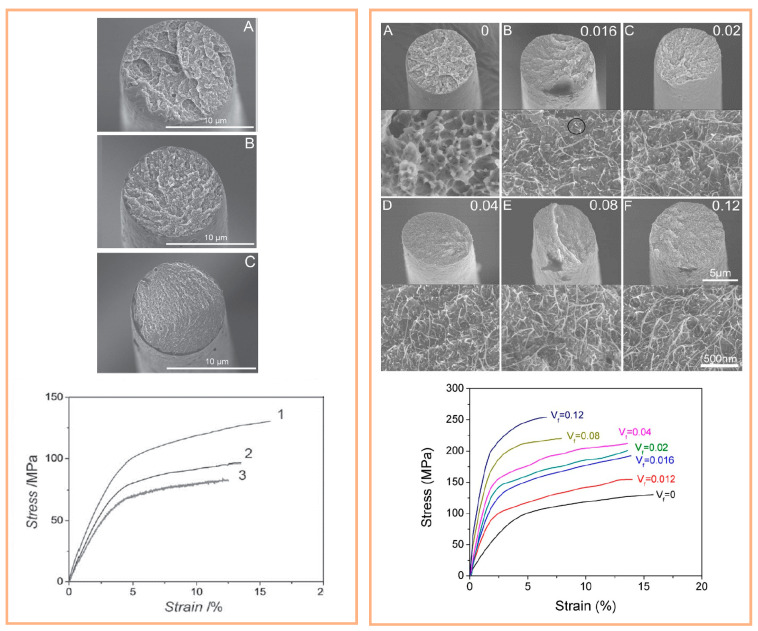
(**Left**) Representative SEM images of PEDOT:PSS fiber spun into (A) acetone, (B) IPA, and (C) PEDOT:PSS–PEG fiber spun into IPA. Representative stress–strain curves for (1) PEDOT:PSS fiber prepared using isopropanol coagulation bath, (2) PEDOT:PSS–PEG fiber spun into IPA, and (3) PEDOT:PSS fiber spun into acetone [67]. (**Right**) SEM images of the cross-sections of PEDOT:PSS/PEG–swCNTs composite fibers broken under tensile strain at low and high magnifications showing shape and microstructure of PEDOT:PSS/PEG–swCNTs composite fiber at various PEG–swCNTs loadings. Representative stress-strain curves of PEDOT:PSS/PEG–swCNTs composite fibers at various nanotube loadings [68].

PEDOT:PSS:PEG fiber was produced by means of an innovative one-step wet spinning process. In order to create a continuous spinning process, the PEG was mixed into spinning mass. Here, isopropanol (IPA) was employed as nonsolvent in the coagulation bath to create a prolonged relaxation time for polymer chains; as a result, a molecular orientation along the fiber axis was established during the spinning process. Thereby enhanced mechanical properties for the wet-spun PEDOT:PSS:PEG fiber (Figure 16, left) [67]. Later, Rouhollah Jalili et al. developed PEDOT:PSS fibers with polyethylene glycol (PEG) and swCNT using wet spinning technology. The introduction of PEG–swCNTs into PEDOT:PSS matrix improves the electrical conductivity and mechanical properties while providing an additional active material for capacitive energy storage (Figure 16, right) [68].

**Figure 17:**
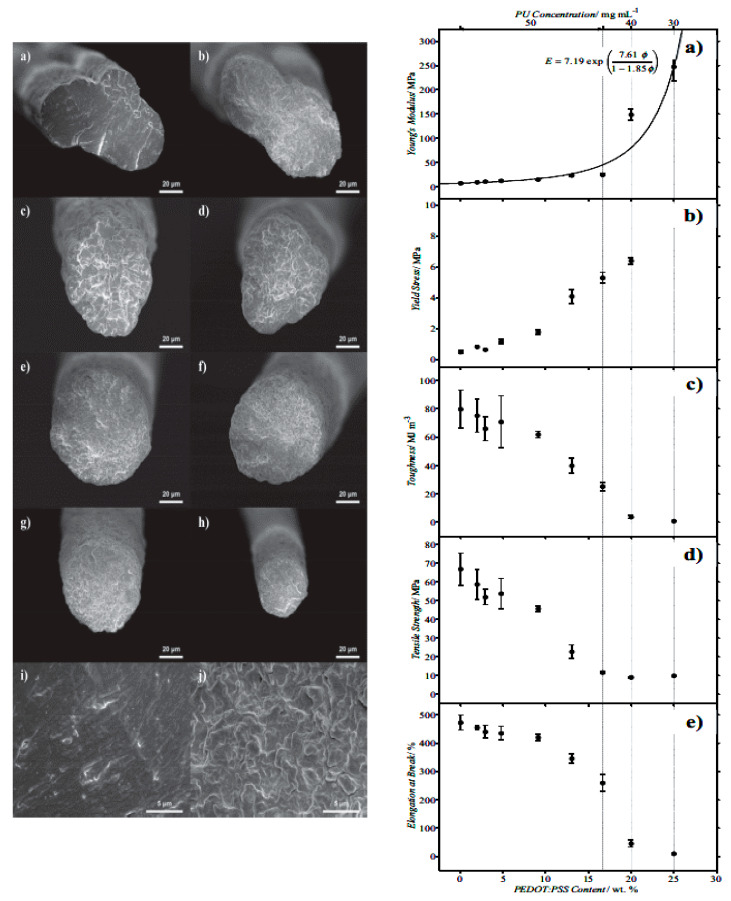
(**Left**) SEM micrographs of fibers produced from spinning formulations in DMSO into a coagulation bath of isopropanol/water (80/20 v/v): (a) pure PU fiber, b–h) PU/PEDOT:PSS fibers with PEDOT:PSS loadings of (b) 2.9 wt%, (c) 4.8 wt.%, (d) 9.1 wt.%, (e) 13.0 wt.%, (f) 16.7 wt.%, (g) 20.0 wt.%, (h) 25.0 wt.%, (i) higher magnification of (a), and (j) higher magnification of (e). (**Right**) Mechanical properties of PU/PEDOT:PSS fibers at different PEDOT:PSS content: (a) Young’s modulus, (b) yield stress, (c) toughness, (d) tensile strength, and (e) elongation at break [69].

**Figure 18:**
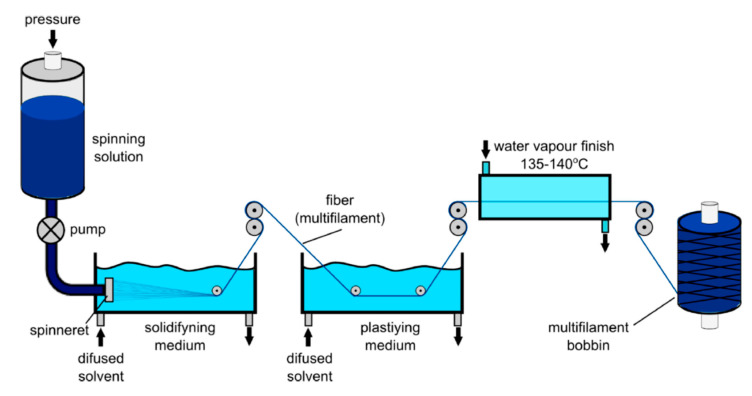
Scheme of fiber spinning using the wet spinning [79].

Although fibers made from ICPs possess great conducting properties and tensile strength, they also exhibit relatively low to no elasticity or formability and cannot be elongated by more than 20% of their original length. However, the combination of high elongation, conductivity, and tensile strength is essential for potential application scenarios that often involve high elongations. A fiber based on PU and a PEDOT:PSS solution was produced via wet spinning (Figure 17, left). A higher Young´s modulus was determined for PU/PEDOT:PSS fibers compared to pure PU fibers, while also providing less tensile strength and elongation at break (Figure 17, right). Once the amount of PEDOT:PSS was increased in a PU fiber, the Young´s modulus increased exponentially (Figure 18, right). The tensile strength behavior can be explained by the structure of polymer chains containing soft as well as hard PU segments. Soft segments in the PU network were interfered with by adding PEDOT:PSS, causing the fiber to break quickly and have less tensile strength [69].

Wet spinning technology was also employed to produce a core-sheath type of fiber by using PEDOT:PSS as inner core and PPy as electropolymerized outer sheath layer. This type of coaxial conducting polymer fiber was used for antibiotic treatment [70]. Wang et al. also produced PEDOT:PSS by use of wet spinning technology. They selected ethanol/water with calcium chloride (CaCl_2_) for the coagulation bath. The final step involved the immersion of PEDOT:PSS fibers into concentrated sulfuric acid solution to remove insulated PSS. The produced fiber electrode exhibited a high electrical conductivity (1771.8 S/cm) and tensile strength (112.7 MPa) [71].

Moreover, PPy was blended with extrinsically conducting fillers, such as graphene, and graphene–PPy fibers were spun via wet spinning [72]. Foroughi et al. doped PPy with di-(2-ethylhexyl)sulfosuccinate (DEHS). Furthermore, organic solvents, such as NMP, DMSO, dimethylformamide (DMF), and m-cresol, were selected for use in coagulation baths. PPy fibers were manufactured from a PPy–DEHS blend by means of solvent spinning technology. The tensile strength, Young´s modulus, elongation at break, and electrical conductivity were 25 MPa, 1.5 GPa, 2%, and 3 S/cm, respectively [73]. In addition to PPy, intrinsically conducting PAni polymers were also employed for wet spinning. PAni in combination with poly-ω-aminoundecanoyl (PA11) was dissolved in concentrated sulfuric acid; the solution obtained was then processed by wet spinning to generate conducting PAni–PA11 fibers. The resulting electrical conductivity of these fibers was insufficient, i.e., between 10^–6^ and 10^–1^ S/cm [74,75].

To generate silk fibers (SF) with the desired electrical conductivity, a SE solution was blended with conducting anorganic polymers, e.g., PAni, PPy, or PEDOT. Based on these solutions and by use of wet spinning including an aqueous coagulation bath, electrically conducting fibers were produced. With a PAni content of 0.28 wt.%, a high specific resistance of 0.704 × 10^4^ Ω·mm was measured for the investigated SF–PAni filaments. Measurements also yielded a tension and elongation of SF–PAni filaments of 22.08 ± 0.2 MPa and 63.2 ± 2.56%, respectively [76].

An alternative approach for improved mechanical properties of PAni fibers suggested the manufacturing of solutions based on other fiber-forming materials. Hsu et al. [46] described the production of PAni/poly(p-phenylenterephthalamid) fibers using sulfuric acid solutions. The conductivity of these fibers was approx. proportional to the weight concentration of PAni. Experimental results revealed that a fiber with 30 wt.% PAni can achieve a favorable electrical conductivity of 0.1–1.8 S/cm. The corresponding strength, module, and elongation at break were 15 gpd, 300 gpd, and 4%, respectively. Similar fibers were manufactured by wet spinning and the use of sulfonated PAni [77]. Jiang et al. reported on the wet spinning of PAni/polyacrylonitrile-methyl acrylate (Co–PAN) fibers. The fibers obtained with a PAni content of 7% provided a relatively low electrical conductivtity of 10^−3^ S/cm due to dodecylbenzenesulphonic acid (DBSA) doping [78]. Karbownik et al. used wet spinning (Figure 17) to produce PAN/PAni in-situ fibers; the generated fibers were characterized by their black color, an electrical resistance of 5.47 kΩg/cm^2^, and low mechanical strength [79].

PEDOT, PAni, and PPy were among the most frequently used ICPs for wet spinning processes (Figure 18). PS or PA11 were typically employed as organic dielectric materials, whereas sulfonic acid was often used as doping agent. Coagulation baths contained acetone or EG, which in many cases were suitable for post-treatment purposes as well. Most wet spinning technologies require several process steps, prolonged relaxation periods, and added extrinsically conducting fillers, e.g., swCNT and mwCNT. A fiber combining low electrical conductivity, high tensile strength, and high elongation has not yet been produced. In conclusion, it is essential to adjust and further investigate all essential spinning material properties (viscosity, conductivity, surface tension, blending behavior, concentration of coagulation bath), process parameters (temperature of spinning material and coagulation bath, yarn take-off speed, processing time), process steps (washing, drawing, drying, post-treatment), and the doping of ICPs according to polymer type in order to achieve fibers with the desired electrical conductivity, high strength, and high elongation.

#### 4.2.3. Melt Spinning

From an industrial perspective, melt spinning is to be preferred over wet spinning, since it does not require a coagulation bath nor washing processes and can operate on a higher production speed as well as allows the use of different thermoplastic polymers [80]. To produce a fiber based on ICPs with a homogeneous conductivity over its entire length, the spinning material without percolation threshold should also be homogeneous and conducting. Although the production of this fiber type is very complicated and delicate based on melt spinning technology, it is nevertheless a promising topic with melt spinning being the most frequently used process for the manufacturing of continuous textile fibers.

Kim et al. introduced melt-spun fibers made of PAni/polypropylene (PP) blends; dodecylbenzenesulphonic acid (DBSA) was used as doping agent. The electrical conductivity of the spun fibers was approx. 10^−9^ S/cm, with a PAni concentration ranging from 1% to 40%. This team of researchers identified the non-homogeneous distribution of PAni within the PAni/PP fiber as reason for its low conductivity [81].

PAni itself is infusible, though can be processed under specific thermal conditions. For example, Azadeh Soroudi et al. doped PAni with dodecylbenzenesulfonic acid, mixed it with PP, and subsequently used melt spinning to generate matrix–fibril fibers made of PAni/PP. The electrical conductivity of PAni/PP fibers was insufficient as well, again most likely due to lacking homogeneity. To address this issue, adverse fibril formations (droplets or vertical stripes) in the matrix (PP) were increased, and fibril breakage (no excessive tension) was prevented. Fibril formation also depends on the viscosity of the matrix; if PP provides a high viscosity, fibril structures are shaped in the form of droplets instead of vertical stripes, thus enabling an advantageous conductivity level. The maximum conductivity of fibers that were drawn four times was 10^−4^ S/cm—a value that is still insufficient. The corresponding strength, Young´s modulus, and elongation at break were 1.53 cN/tex, 3.05 cN/tex, and 481.8%, respectively [82]. It should be noted that PAni does not belong to the class of thermoplastics so that crosslinking and degradation reactions occur in the PAni complex once 200 °C is exceeded. Therefore, PAni is generally processed as part of a blend with thermoplastic polymers characterized by low melting points (<200 °C) in order to suppress potential side reactions by PAni. PP with its melting temperature of 160 °C is particularly suitable for the melt spinning process in combination with PAni [81,82,83].

Fanous et al. produced an electrically conducting fiber from P3HT without thermoplastic polymers by means of melt spinning. Iron (III) chloride (FeCl_3_) in nitromethane served as doping agent. The researchers reported that electronic transport, and therefore conductivity, of spun fibers was increased once the degree of crystallinity rose as well. To increase crystallinity, melt spinning offers the possibility of drawing above Tg and subsequent fiber stretching. Importantly, electro- and wet spinning do not provide this option. The electrical conductivity of P3HT fibers doped with FeCl_3_ was reduced from 360 S/cm to 160 S/cm after stretching. Additionally, the film building properties of P3HT were exploited to create P3HT-coated PET fibers. In this case, the drawing of P3HT-coated fibers led to an increase in crystallinity in the P3HT coating and electrical conductivity [84]. The high level of electrical conductivity was achieved by longitudinal fiber stretching; during this process, the polymer chain was oriented in the longitudinal direction so that well-structured molecules and a higher degree of crystallization was obtained. In conclusion, the electrical conductivity and charge carrier mobility of the produced fibers were improved.

Some of the first reports on the melt processing of poly(alkylthiophene)s date back to the 1980′s when Yoshino et al. prepared a flexible conductive polymer fiber by melt-spinning of poly(3-alkylthiophene)s [85] however without providing information on their structure and crystallinity. Another method for increased crystallinity and thus enhanced conductivity of coatings based on P3HT involves tempering above Tg. Thermal extrusion of PAni is possible by blending with PP [86]. A flexible hollow fiber was obtained through the combination of PAni with previously spun feixu (from the seeds of Cynanchum, reed, and endives) by means of in-situ polymerization, thus generating a flexible PAni/feixu composite fiber [87]. Intrinsically conducting polymers were spun with or without thermoplastic polymers using melt spinning. In most cases, PP was used as thermoplastic component due to its low melting temperature being beneficial for the melt spinning process. Dodecylbenzenesulfonic acid (DBSA) and FeCl_3_ in nitromethane were often used as doping agents, whereas PAni or P3HT were selected as ICPs.

The most commonly used ICP for spinning have so far been PAni, PEDOT, PPy, and P3HT (Table 3). By adding a second polymer, such as polyethylene oxide (PEO), PSS, PMMA, PC, or PVA (Table 3), to the spinning solution, ICPs can be spun to fibers. PAni was typically mixed with PEO, polylactic acid, PVP, PMMA, polyvinylidene fluoride (PVF), PC, and/or PS. PMMA, PVF, or PS were often used as organic dielectric components, whereas glycerol, sorbitol, DMSO, NMP, or EG were selected as doping agents. Fibers made from ICP based on electro-, wet, or melt spinning exhibited different electrical conductivities (Table 3).

PAni, P3HT, and PT were among the most frequently used ICPs combined with PP or PET for melt spinning processes. Presently, there is no scientific literature addressing the development and manufacturing of polyamide (PA), polyether ether ketone (PEEK), or PI composites with other ICPs, such as PEDOT, or PPy, or in combination with ICPs with extrinsically conducting fillers, e.g., carbon black, swCNT, or mwCNT. To achieve fibers with a homogeneous electrical conductivity based on ICPs, potentially in combination with extrinsically conducting fillers, future research efforts should be directed to the defined adjustment of the amount of ICPs in thermoplastic polymers, the conservation of viscosity and conductive properties, the percolation threshold of extrinsically conducting fillers, and the integration of intrinsic polymers into PA, PE, or PEEK composites. So far, no research projects have been dedicated to the compounding of thermoplastic polymers with ICPs and/or extrinsically conducting fillers.

Furthermore, additional research is required to complete systematic rheological, thermal, experimental, and analytical investigations as well as conductivity analyses in order to identify masterbatch formulations, which can then be melted in an extruder, granulated, doped, and spun. In addition to the processes of melting, extrusion, and forming in the spin pack, it is essential to evaluate the influence of drawing on spun electrically conducting fibers, based on which the geometry (yarn diameter) as well as textile-physical and electrical properties (tensile strength, yield strength, percolation threshold, conductivity) can be modified in terms of different textile-technological processing technologies (knitting, weaving). Hence, decisive process parameters, e.g., temperature profile, throughput, and take-off and winding speeds, must be determined to enable the manufacturing of fibers with defined properties using the complex melt spinning process.

### 4.3. Finishing and Coating

As discussed in Section 4.2, yarns manufactured from ICPs possess favorable electrical conductivities, but low mechanical properties, thus making them a challenging option in terms of smart textiles. For the production of textile yarns with desirable mechanical and electrical properties using ICPs, a promising option involves the coating of standard textile yarns with a layer of ICPs. So far, various ICPs, such as PAni, PPy, and PEDOT:PSS, were used in coating layers. Moreover, natural fibers, e.g., CO, WO, and silk (SE), as well as chemical fibers, e.g., PET, PP, PA, PAN, and PU, were used as substrates. For the application of ICP layers to textile materials, two main coating technologies were developed, i.e., the dipping and drying method and the chemical solution/vapor polymerization method.

#### 4.3.1. Dipping-and-Drying Technique

The dipping-and-drying technique is the simplest method for producing conducting textile yarns or fabrics; it involves the dipping of textile material surfaces into conductive solution. During the drying process, the solvent evaporates so that a conductive layer is deposited on the yarn surface. To achieve an increased conductivity, this dipping and drying process can be repeated multiple times. Alternative coating techniques, such as hand brushing and spraying, are considered to be variants of the dipping-and-drying technique. These approaches are relatively straightforward and do not require cost-intensive devices or complex processing steps. Kim et al. coated PP and PET yarns with a PAni solution doped with dodecylbenzenesulfonic acid (DBSA) using the dipping technique; they reported on PET yarns with an enhanced Young’s modulus and tensile strength. This positive result can most likely be attributed to the effect of PAni-conducting layers on fiber-to-fiber areas of the PET yarn in addition to decreased friction between fibers due to the smooth surface of the intrinsically conducting layer [81]. The proton in the acid reacted with the imine in PAni, leading to protonation and conductivity. The electrical resistance of these coated yarns ranged from 10^3^ Ω and 10^6^ Ω, which is insufficient for the production of highly electrically conducting layers. Furthermore, the PAni-coated PET fiber was able to maintain its strength and flexibility. Previous research by Nouri et al. had suggested that the PAni coating might have an “insignificant” effect on the mechanical properties of PA, WO, CO, and PET yarns [88].

To meet the challenges that occur during dip coating to deposit ICP onto textiles, alternative approaches including in-situ polymerization were investigated [89]. In the case of this research project, the ICP was physically absorbed at the textile surface, and subsequently polymerized along the solid/liquid interface to achieve a thin, yet homogeneously conductive layer at the fiber surface [90]. Yue et al. evaluated this coating technique by deposition of PPy on a Nylon–Lycra^®^ fabric, whereby an oxidizing and doping agent was used to enable the polymerization of monomers. Thus, a conducting PPy layer on all fibers with a surface resistance of 149 Ω/square [91] was realized. The Nylon–Lycra^®^ fabric was coated without current with nickel/phosphorus and nickel/PPy to manufacture a flexible and electrically conducting fabric. It was observed that the surface resistance of the coated fabric decreased over prolonged periods of nickel deposition [92].

**Figure 19:**
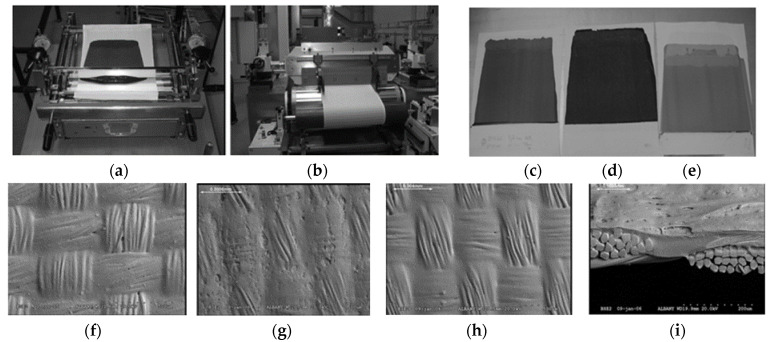
(**a**) A knife-over-roll lab coater. (**b**) Large scale continuous knife-over-roll coater. (**c**–**e**) Laboratory coated fabrics. (**c**) PAni, (**d**) PPy, and (**e**) PT. (**f**–**i**) SEM micrographs of the coated fabrics. (**f**) PAni, (**g**) PPy, (**h**) PT, and (**i**) cross-section of fabric coated with PPy [95].

R. Neelakandan et al. coated a PES fabric with PAni by means of in-situ polymerization at room temperature (30 °C) for a duration of approx. 2 h while stirring occasionally. Hydrochloric acid was selected as doping agent and ammonium persulphate was used as oxidizing agent. PES fabrics with varying binding patterns, e.g., plain weave, twill weave, and satin weave, were used. The specific resistance of the PET satin fabric was higher compared to plain and twill weaves, as the satin weave contained fewer intersections, and PAni-coated filaments were in close contact with adjacent filaments. Furthermore, R. Neelakandan et al. stated that the favorable electrical conductivity and the homogeneous layer absorption depended on the fabric structure, binding pattern, and areal weight [93]. In addition, ICP layers may oxidize when exposed to air, which can cause damage and decrease conductivity. Therefore, Wu et al. suggested the storing of yarns coated with ICP in a desiccator [94]. Rehnby et al. coated PET fabrics with a conducting coating paste by means of the knife-over-roll technique (Figure 19a,b). PAni, PPy, and PT including an acylate binder were used as ICPs. This team of researchers stated that when loading was applied to the fabric, its multi-layer structure caused irregularities (Figure 19c–i). This led to uneven spreading of the coating layer on the PET fabric surface. The surface resistance was measured as 3.8 × 10^12^ Ω for samples treated with 1.3 wt.% of PT [95].

Irwin et al. reported on an SE fiber that was dipped in PEDOT:PSS/EG solution. They found that the resulting electrical conductivity of 8.5 S/cm was ten times lower than that of Ag-coated yarn [96]. Varesano et al. coated CO with PPy via dipping to generate antibacterial properties. Two different doping agents, such as chloride and dicyclohexyl sodium sulfosuccinate, were used for this purpose. The antibacterial effect of PPy is assumed to be caused by positive charges in the polymer chain interacting with bacterial cell walls, thus causing it to break [97]. Ikma Omar et al. coated CO and PET fabrics with PAni to generate an antibacterial effect using the dipping technique and an immersion time of 30 min; phytic acid (10%, 20%, and 30% (*v*/*v*)) was selected as doping agent. During doping with 30% (*v*/*v*) phytic acid, optimal conductivities of 2.28 × 10^−4^ S/m (CO), and 2.15 × 10^−2^ S/m (PES) were measured. Tests revealed that the produced PET fabric possessed a relatively high antibacterial activity in direct comparison with CO towards *K. pneumoniae*, *S. aureus*, and *E. coli* strains [98].

Several shielding applications for the protection of human health and electronic devices from the dangerous effects of electromagnetic radiation require new solution approaches. J Avloni et al. discovered a correlation between shielding efficiency and the surface conductivity of PPy-coated PET nonwovens. The samples with the lowest surface resistance values provided a great electromagnetic shielding effect of 37 dB [99]. Sparavigna et al. investigated a heat generation process for heated textiles based on a PET fabric coated with anthraquinone-2-sulfonic acid-doped PPy [100]. Furthermore, as described by Sakthivel et al., CO fabric was coated with PAni using the dipping technique and subsequently dried at 50 °C. In this research project, ammonium persulphate was used as oxidizing agent [101]. Rebelo et al. introduced the coating of bacterial cellulose (BC) fibers with a double layer comprised of poly(4-vinylaniline) (PVAN) and PAni; an electrical conductivity of 4.5  ±  1.7 ×10^−2^ S/cm was measured [102]. Akerfeldt et al. coated PET fabrics—first with PU, and subsequently with PEDOT:PSS/EG. The researchers described that EG had the most significant influence on electrical conductivity, tear resistance, and bending stiffness of the coated textile fabrics [103]. By the coating of WO yarns using PPy [104,105], it was shown that the viscosity and elongation at break of yarns can be increased to 7% and 21%, respectively.

The research team of Merlini et al. coated amorphous silica short fibers (ASF) with PPy and polystyrene-block-poly(ethylene-ran-butylene)-block-polystyrene (SEBS). FeCl_3_ was selected as oxidant. The high fiber length/fiber diameter ratio of PPy/ASF fibers in addition to their beneficial electrical conductivity make them suitable for the production of conducting polymer composites at a low percolation threshold [106]. Trifigny et al. employed the piezoresistive characteristic of a developed sensor thread made from PEDOT:PSS-coated e-glass fiber rovings for the in-situ monitoring of strains and stresses acting on yarn during the weaving process. PEDOT:PSS was doped with NMP. Polyvinylalcohol (PVA) was utilized in order to improve the mechanical properties of the sensor film. After applying six coatings, the sensor yarn offered a satisfying average resistance value of 100 kΩ [107]. Eom et al. developed a strain sensor by use of PEDOT-coated PES fibers [108]; these fibers were coated in a PEDOT solution for 20 min at 70 °C. Hence, a PEDOT layer thickness of 100–300 nm providing an electrical resistance of 600 Ω/cm was achieved. Compared with uncoated PES fibers, the Young’s modulus of coated fibers was higher. Electro-spun PET nanofibers were coated with PEDOT:Tosylat (TS), with TS acting as doping agent. PEDOT/TS is conductive, transparent or bluish, and characterized by its high thermal, photochemical, and hydrolytic stability. The suspension of the polymer as gel particles in water, with PSS acting as doping agent, can be processed very easily and is therefore used for a wide range of applications involving electrode materials for organic building components or antistatic coatings. 3D scaffolds were produced based on nanofibers made from PEDOT:TS to induce Ca^2+^ signals in SH-SY5Y neuroblastoma cells [109]. Firstly, Nuramdhani et al. applied a hydrophobe coating, i.e., a fluorocarbon and thermoplastic polyurethane (TPU) solution, and secondly, a conducting PEDOT:PSS solution to a PET:CO blended textile fabric [110]. The PEDOT:PSS layer that is located between two conducting yarns (electrodes) shows a capacitive behavior in textile energy storage systems. Various commercially available PEDOT:PSS yarns were selected as electrolytes, e.g., Clevios P-VP-AI-4083 and Orgacon ICP 1050, whereas stainless steel and silver-coated polybenzoxazole (Ag/PBO) were used as electrode. The thermal stability of PEDOT:PSS was validated up to 250 °C. PEDOT:PSS with a lower conductivity yielded beneficial results in terms of charge–discharge cycles and capacitive behavior [110]. PEDOT is very stable and suitable for integration into finishing liquors. In contrast, PAni is highly sensitive, which may be an obstacle when generating formulations. Hence, thickeners were added to finishing liquors. Electrically conducting polymers were applied onto textile materials, e.g., PES, in the form of an aqueous dispersion, using the Foulard or padding technique as well as spray coating [111]. Electro-spun PU microfibers were coated with a layer containing conductive PAni. A sheet resistance (length, width, thickness of approx. 1 cm, 5 mm, and 100 µm, respectively) of 2 kΩ and a conductivity of 10 S/cm were measured [112]. Once textile fabrics were treated with the conducting polymer PPy, a thin electrically conducting layer was generated on the membrane so that the formation of biofilms could be avoided or at least reduced and the effect of biocides enhanced. An electrical resistance of 0,4 Ω was determined for coated filtration membranes (0,22 µm) made of PVDF and PES [113]. While a standard synthetic fiber provides a surface resistance of approx. 10^9^–10^5^ Ω, this value was lowered to 1–10^2^ Ω by the application of a conductive polymer coating.

One of the most widely used methods for polymer modification involves the combination of two or more materials with differing characteristics. The blending of polymers is a common method for the production of innovative polymer materials with customized properties without the need for synthesizing completely new materials. For example, to improve their structural, optical, and electrical properties, ICPs were combined with extrinsically conducting fillers.

PES was coated with PPy to create a conducting textile fabric. Hence, the extrinsically conducting filler graphene oxide (GO) was employed as counter ion with different concentrations (10%, 20%, and 30 wt.%) in order to neutralize/dope the positive charges of the PPy structure. The doping degree (N+/N) decreased with rising GO concentrations. Conductivity was modified by adjusting the GO content, whereas conductivity and electroactivity both decreased once the GO content was increased [114]. Another research report presented the application of reduced graphene oxide (RGO) and PPy onto CO fabrics by means of the thermal reduction of GO and thermochemical polymerization of pyrrole. The resulting PPy–RGO fabric exhibited a high electrical conductivity of 1.2 S/cm [115]. Additionally, PAni/APTES/silicon carbide nanocomposites with a core-sheath structure were successfully manufactured by using a simple in-situ polymerization process. The functionalization of silicon carbide with aminosilane contributed positively to a homogeneous envelope curve and thickness of the PAni layer on silicon carbide, which is essential for the desired impedance and damping coefficient. Results revealed that these unique nanocomposites fulfill extensive requirements in terms of both efficiency and lightweight engineering [116].

For the application of coating or aqueous dispersions of ICPs such as PEDOT:PSS with carbon black, onto PET fabrics, standard techniques can be used, including application with a scraper or dipping into solutions. In this case, carbon black for enhanced conductivity and sodium dodecylsulfate as anionic surfactants were applied via the dipping method [117].

Electrical conductivity reacts very sensitively to changes in material volume due to expansion if the content of extrinsically conducting fillers is close to, yet above the percolation concentration. Even minor volume changes interrupt many conducting paths, thus generating significant variations in the total resistance of sensor materials. Hence, it must be noted that the conductivity of coated yarns depends on the homogeneous distribution of conduction material in solutions.

However, stable electrically conducting textile materials with continuous mechanical properties can be realized by means of the dipping-and-drying technique. There are various influencing factors for the coating process, e.g., the type of ICP (PAni, PEDOT, PPy, or PT), the type of textile yarn (CO, WO, PET, PA, PP, etc.), the binding pattern (twill weave, satin weave, plain weave), the area mass (60 g/m^2^–300 g/m^2^) of the fabric, and the twist density of the textile yarn. Other decisive factors include the concentration of ICPs and doping/oxidizing agents, the ratio of binding, oxidizing, and doping agents and ICPs, the pH value, and the viscosity of the solution. To achieve the desired level of electrical conductivity, textile materials were coated multiple times. Thus, the stability of the layer improved, while its homogeneity and adhesion properties between individual layers decreased.

#### 4.3.2. Chemical Solution/Vapor Polymerization

**Figure 20:**
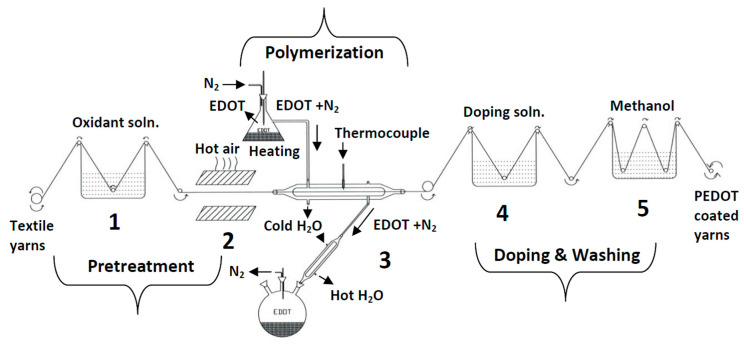
Schematic diagram of the CVD process for the production of PEDOT-coated conductive yarns [118,119,120].

**Figure 21:**
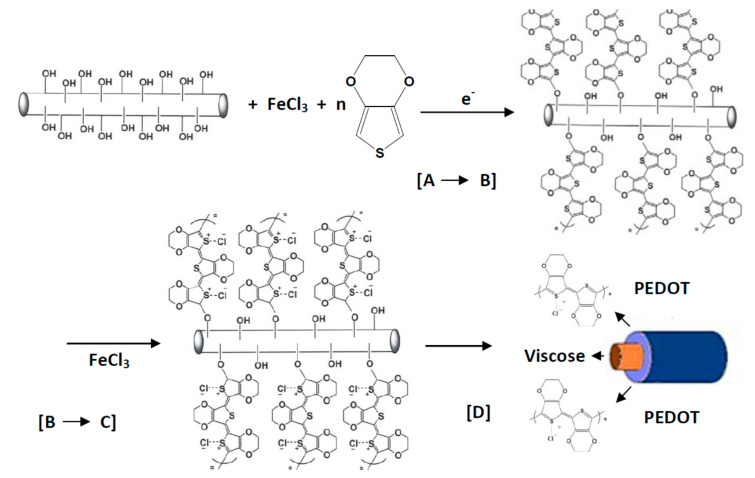
CVD-based mechanism for polymerization of PEDOT on the surface of viscose fibers (**A**→**B**) oxidation of EDOT monomer to PEDOT, (**B**→**C**) doping of PEDOT, (**D**) sketch of PEDOT-coated viscose fiber [119].

This section will focus on recent developments in terms of coating application techniques and processes for ICPs on textile materials. In addition to the dipping-and-drying method, a coating of ICPs can also be applied by means of chemical solutions and vapor polymerization. Chemical polymerization is typically performed by the pre-treatment of substrate yarn using an oxidizing agent (e.g., FeCl_3_). Polymerization is initiated via monomer oxidation forming radical cations; they are subsequently linked to form insoluble oligomers and polymers that deposit on the surface of substrate yarns. In general, it can be stated that the chemical polymerization of ICPs on textile yarn generated a homogeneous layer with a higher conductivity compared to material coated via dipping and drying. Bashir et al. introduced oxidative chemical vapor deposition (oCVD) for the deposition of PEDOT on viscose (CV) and PES yarns (Figure 20) [118,119,120]. The mechanism of formation of PEDOT on the surface of CV fibers and the step involving doping PEDOT polymer chains with FeCl_3_ dopant are shown in Figure 21 (A→B and B→C, respectively). By an increase in oxidizing agent content (FeCl_3_), conductive yarns were produced. However, thicker PEDOT layers on CV fibers decreased their mechanical properties. To overcome this issue, PET was investigated in terms of its potential as alternative substrate for the oCVD of PEDOT, in particular due to its temperature resistance, favorable mechanical properties, and chemical stability when exposed to acidic environments (FeCl_3_ oxidizing agent). PEDOT-coated PET fibers offered an improved electrical conductivity in addition to better mechanical properties compared with PEDOT-coated CV fibers [121]. Fairbanks et al. coated different fabrics (CO, SE, PET) with PEDOT using oCVD [122]. Xue and Tao applied PPy onto the polycaprolactam (PA6) and PU (Lycra^®^) fiber surface by oCVD; FeCl_3_ × 6H_2_O was used as oxidizing agent [123]. Radoičić et al. coated a PET fabric with a PAni/TiO_2_ solution by use of chemical oxidative polymerization, whereas ammonium persulfate was selected as oxidizing agent. The presence of TiO_2_ nanoparticles affected the dielectric properties (permittivity and dissipation factor) and the alternating current conductivity of PAni/TiO_2_ coated PET fabrics. Thus, PET fabrics equipped with a PAni/TiO_2_ coating provided a higher level of conductivity as compared to PAni-coated PET fabrics [124].

Another very promising approach is the use of an already developed vapor phase polymerization (VPP) technique for coating of textile materials with conjugated polymers, to obtain highly conductive textile fibers and yarns [125,126]. Based on this technique, conducting fibers with favorable mechanical as well as electrical properties can be generated. Furthermore, Vapor Polymerization Deposition (VPD) techniques enable the formation of a homogeneous layer by addition of monomers to the substrate surface during the vapor phase. These monomers are either self-initiating or introduce a second initiating species to the polymerization process in order to generate a polymer film. The process speed can be adjusted by lowering the substrate temperature of ICPs and other additives, thus making it a suitable option for temperature-sensitive textile substrates. VPD techniques offer various advantages for the processing of ICPs: for example, the deposition does not depend on the substrate, coatings are adaptable, and it is suitable even for ICP that are difficult to process as solutions. A challenge is posed by the even deposition of monomers and oxidizing agent in the case of large-size substrates [125,126]. Laforgue et al. treated CO and PET textile fibers with an iron p-toluenesulfonate oxidizing solution, which were then dried. The alkalinity of the solution was modified via pyridine. Subsequently, the treated fibers were coated with PEDOT by means of the vapor phase technique to achieve conductivity. The resulting fibers were characterized by a high electrical conductivity of 10 S/cm [127]. Bashir et al. discovered that the electrical properties of PEDOT-coated CV fibers strongly depend on the concentration of oxidizing agent (FeCl_3_) and the doping process of PEDOT. Fiber resistance measurements prior to and after doping proved that fibers with high concentrations of oxidizing agent also provided the highest conductivity values. Long-term current flow through the fibers showed that no charging effect occurred. These fibers are suitable for a large variety of applications in electronics, where a constant conductivity over a longer period of time is required [118,119,120].

**Figure 22:**
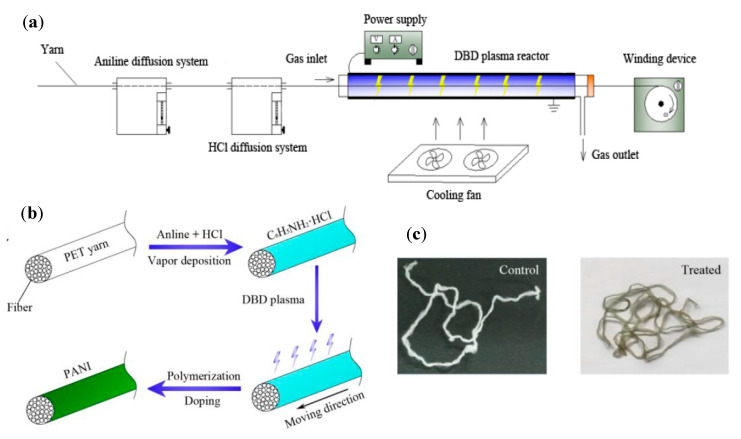
(**a**) Experimental device of vapor phase in situ polymerization of PAni; (**b**) Illustration of the treatment procedure; (**c**) chemical structures of idealized oxidation states of PAni [37].

Liu et al. introduced a conducting PET yarn that was doped with aniline and hydrochloric acid via VDP, coated with PAni, and produced by means of Dielectric Barrier Discharge (DBD) at atmospheric pressure (Figure 22). The PAni-coated PET yarn offered excellent electrical resistance properties of 0.045 MΩ·cm after DBD treatment [38].

Isabel et al. produced textile sensors made from PES and PEDOT for the continuous monitoring of electrocardiograms (ECG); they used in-situ vapor phase polymerization (VPP) techniques combined with aqueous oxidizing solutions (FeCl_3_). Their research efforts revealed that textile electrodes have a great potential for replacing standard metal electrodes, although their efficiency still requires optimization in terms of fiber modifications, surface morphology, and electrical conductivity [128].

WO yarn was coated with PPy based on a continuous vapor polymerization technology using different FeCl_3_ concentrations as oxidizing agents. Optimum specific electrical resistances were measured at 2.96 Ωg/cm^2^ at 80 g/l FeCl_3_ and 1.69 Ωg/cm^2^ at 70 g/l FeCl_3_ [129]. After pretreatment and activation steps, Hosseini et al. coated CO and SE with PPy by use of two different methods: vapor phase and liquid phase. This team of researchers suggested to use vapor phase coating, since gas penetrating the PPy layer was expected to yield fibers with enhanced adhesion. However, an uneven PPy layer was found on the CO and SE fiber surface. Electrical conductivities of 6.4 × 10^–4^ S/cm for CO and 3.2 × 10^–4^ S/cm for SE were measured [130]. Tsukada et al. reported on the electrochemical coating of SE fiber bundles using a PEDOT:PSS solution. The addition of glycerol to the PEDOT:PSS–SE fiber bundle enhanced its electrical conductivity from 20.6 × 10^3^ S/cm to 0.102 S/cm [131]. The conductivity of WO and PES yarns with a PAni coating was significantly lower than that of CO, PAN, and PA [105].

The surface resistance of PET fibers that were electrochemically coated with PAni (2 wt.%) was 3 × 10^5^ Ω/square [132], whereas PPy-coated fibers possessed resistances of up to 10 Ω/square [133]. Unfortunately, the use of PEDOT/PSS is currently very limited due to its brittleness and undesirable processing properties. A flexible and stretchable sensor material was generated from a thin film containing elastomeric PU (Lycra^®^), thermoelectrical PEDOT/PSS, and ethylene glycol (EG) [94].

Qi Xua et al. used PPy/TiO_2_ for the coating of CO fabrics by means of the sol-gel process as well as in-situ polymerization. Based on these fabrics, flexible electrodes for super capacitors were manufactured [134]. Wu et al. applied a coating containing poly(2-methoxyaniline-5-sulfonic acid)-doped PAni to WO fibers using an electrochemical approach [135]. Grancarici et al. produced sensor yarns from E-glass/PPy yarn using a PEDOT:PSS solution and roll-to-roll coating to enable the monitoring of composite structures [136]. Varesano et al. investigated the thermal stability and flame resistance of PPy-coated PET nonwovens [137]. In most cases, FeCl_3_ was selected as oxidizing agent and 2,6-naphthalene disulfonic acid as well as disodium salt were employed as doping agents. The surface resistance at a measuring voltage of 10 M for PPy was 178.9 Ω with oxidizing agent, and 100.6 Ω with oxidizing and doping agent. These values can be explained by the doping agent significantly increasing conductivity, which in turn reduces the resistance. PPy-coated PET nonwovens were able to retain their shape after 30 min at 280 °C in a convection oven, whereas uncoated PET melted under identical conditions. The flame resistance was evaluated according to the standardized testing method EN ISO 15025. Untreated PET nonwovens melted instantly when exposed to flames; however, PPy-coated PET did not [137]. According to the state of the art, the main purpose of PEDOT:PSS in standard organic solar cells and light-emitting diodes is to transport charges towards the outside of the layer, as it acts as interface within a sandwich structure between two working electrodes. PEDOT:PSS films possess different conductivities dependent on the transport direction of charges and therefore provide anisotropic conductivity [138].

It is an extremely challenging task to generate an electrically conducting textile material made of ICPs with proper adhesion to the substrate layer and stable mechanical properties based on a Chemical Vapor Deposition (CVD) process. During the application of thin, flexible, conducting layers onto textile fabric surfaces, it is essential to ensure that there is sufficient adhesion between electrically conducting layer and substrate. To improve adhesive properties, the textile material is to be pretreated prior to the CVD procedure using wet-chemical or physical methods. Another disadvantage of the CVD approach is the corresponding aggressive process conditions, i.e., high temperatures (exceeding 200 °C), that are required to create a stable layer with good adhesion. These temperatures pose a considerable problem for the processing of textile materials on an organic basis. Intersections of yarns are potential weak points for the realization of a homogeneous, closed conducting layer. Firstly, due to its curved surface, every fiber has an area at its lower surface where no or only very thin layers can be deposited. Secondly, air gaps emerge between adjacent yarns depending on the yarn fineness and the type of yarn intersections. Therefore, most researchers assume that oxidation occurs on the substrate of in-situ coating and intrinsically conducting materials (monomers) so that the processing temperature can be lowered during CVD coating. Another difficult factor is the undesirable stability of ICPs in solvents or at high temperatures required for drying/curing. These drawbacks have so far limited the application potential of solvent coating techniques, making the vapor phase polymerization technique the more frequently used option. A continuous film formation on a substrate can be achieved by means of oxidative CVD techniques: hence, oxidizing agent and monomer are not applied concomitantly, but the oxidant is applied first via dip coating, followed by the application of ICPs via CVD technology. However, this approach makes it impossible to perform coating with ICPs in a single process step.

Nevertheless, electrically conducting layers can be modified in their thickness by the modification of pH value, temperature, the concentrations of oxidizing agent/intrinsically conducting polymer, deposition speed and period, and the applied electric field; thus, the electrical resistance of the conducting material can be specifically varied. The electrical conductivity of textile yarns and fabrics coated with intrinsically conductive polymers are summarized in Table 4. 

## 5. Applications

Since the discovery of ICPs, their application range has been extended continuously, including electronic components, fuses, sensors, batteries, or corrosion protection systems. Conjugated polymers are of great interest for applications in the fields of data storage, optical signal processing, electromagnetic interference (EMI), and the conversion of solar energy as well as rechargeable batteries, light-emitting diodes, field-effect transistors, circuit boards, and photovoltaics.

### 5.1. Electronic Components

**Figure 23:**
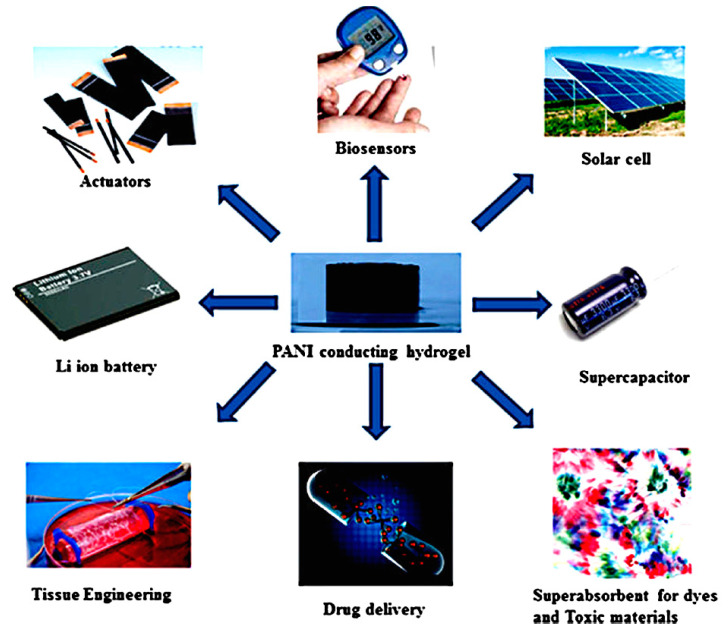
PAni based conducting hydrogels in various potential applications [139].

For electronic components, ICPs are used, for example, in light-emitting diodes (LED), active components in field-effect transistors, and complete or partial organic transistors. Further application scenarios involve photovoltaics and microelectronics, where these polymers are employed, e.g., in electrical connections, for protection against electrostatic charging and electromagnetic shielding [7,22]. PT materials are well-known for their electrical conductivity and optical properties, making them suitable candidates for electrochromic displays, smart windows, photoresistors, and solar cells. PAni-based conducting polymers (Figure 23) can also be printed as micropatterns using inkjet or screen printing. So far, they have been used as part of the flexible electrode for super-capacitors, lithium-ion batteries, and solar cells [139,140].

#### 5.1.1. Batteries and Accumulators

Electrically conducting polymers, such as PPy or PAc, are particularly suitable for batteries and accumulators due to their light weight, high performance capacity, and potentially low manufacturing costs in comparison with currently used metals [1,2,3,4]. For small installation spaces, for example MP3 players or mobile phones, conducting polymers are an appropriate option, as they are not toxic; for the same reason, PPy are also utilized in microbiology and sensor technology. Often times, batteries and accumulators based on polymers have relatively short operational lifetimes, since the charging and discharging cycles cannot be repeated a sufficient number of times. However, due to intensive research activities in the electromobility sector, polymer-based batteries and accumulators are still being investigated by numerous scientists [1,2,3,4].

#### 5.1.2. Light-Emitting Diodes

Intrinsically electrically conducting polymers play an essential role in the field of light-emitting diodes (LEDs). The structure of an LED comprises at least an anode, which is typically transparent and often consists of indium tin oxide (ITO), a thin film of an ICP (e.g., PPV), and a cathode. By applying a voltage, holes and electrodes are generated through anode and cathode, respectively, which then start moving toward one another. Once electrodes and holes meet, they release energy in the form of radiation. Current research projects are focusing in particular on the development of flexible large-scale colored screens [1,2,3]. Doped PT is often used for electronic components including organic light-emitting diodes. The fluorescent color can be varied by adjusting the voltage applied. PPP is used for field-effect transistors and blue light-emitting diodes; in the case of its main usage scenario being organic light-emitting diodes (OLEDs), PPP acts as blue light emitter with a wavelength of λ_max_ = 449 nm [1]. The first OLEDs based on polymers were manufactured using PPV. In general, polythiophene derivates are suitable for various usages, including electrode materials, conductors or semiconductors in organic transistors, light-emitting components in LEDs, and solar cells, due to their great stability when exposed to oxygen or humidity in a doped as well as undoped state.

#### 5.1.3. Solar Cells

Based on the reversal of the LED working principle, i.e., the irradiation of a polymer layer using light for the generation of electron–hole pairs moving in opposite directions to cathode and anode, an organic solar cell can be created producing electricity from light [6,17]. Hence, the structures of LEDs and solar cells are relatively similar. However, in contrast to LEDs, two different conducting polymers are used in a solar cell so that one polymer is responsible for electron transport, while the second polymer is used for hole transport. Often times, an ICP blend containing fullerenes, e.g., PCBM—phenyl-C61-butyric acid methyl ester, is used, since fullerenes prevent the undesirable instant recombination of electron-hole pairs by forwarding electrons to anodes [140].

#### 5.1.4. Smart Windows

Other conducting polymers, such as PAni, can assume various colors due to their numerous modes of oxidation and protonation. These electrochromic characteristics can be exploited for producing smart windows, which are able to switch between colors and absorb sunlight upon voltage application. Advantages over liquid crystals are that these polymers can be produced on a large scale and that there are no limitations in terms of the angle of incident light [13]. For example, thin PPy films are brown to black in an oxidized state, while being yellow to green in a reduced state. Voltage must be applied only for changing the oxidation state.

#### 5.1.5. Supercapacitors

Intrinsically conducting polymers, such as PAni, PPy, PT, and PEDOT including their derivates, are popular materials for stretchable supercapacitors. They not only offer pseudocapacitance due to a redox reaction, but also contribute to the enhanced conductivity of electrodes. They can be used exclusively as electroactive materials or in combination with carbon materials or metal oxides to improve their electrochemical properties. A prestretched styrene-block–isobutylene-block–styrene substrate with a 30 nm gold film and electrolytically deposited PPy was sputtered to manufacture stretchable electrodes with a buckling structure [141]. Hence, the produced supercapacitor was able to retain 81% of its initial capacity. Additionally, PPy and MnO_2_ were galvanically deposited on a carbon nanotube film to build a stretchable asymmetric supercapacitor [142]. PEDOT as well as doped PEDOT were used as transparent synthetic electrode for organically based, optoelectronic applications.

### 5.2. Sensors

Intrinsically conducting polymers, such as PPy, PAni, PT, and their derivates, have been used as active layer in gas sensors since the early 1980s. Due to their unique optical and electrical properties, they offer an excellent application potential for chemo- and biosensors. Gas sensors based on polymers are of particular interest for the monitoring of environmental pollution as these polymers enable rapid changes in conductivity during exposure to vapor, which are generally reversible at room or atmospheric temperatures. Chemoresistors and electrochemical transistors are most frequently used as gas sensors, but in some cases they are also employed as pH sensors, sensors for redox active analytes (e.g., hydrogen peroxide (H_2_O_2_), hydrazine [26]), and in combination with specific analyte receptors (e.g., dopamine [143] or Ca^2+^ ions [144]). Based on their combination with enzymes, these sensors may also act as biosensors [145]. Changes in the physical properties of conducting polymers under the influence of different chemical species make them an attractive option for chemical sensors. The most thoroughly researched conducting polymer is PAni [145,146,147,148], which is sensitive to pH [146], ammonia [146], different alcoholic vapors [147], NOx [148], and H_2_S [149], SO_2_ [149], CO [149], CH_4_ [149], CO_2_ [150] dimethyl methylphosphonate [151], hydrogen halide [151], hydrocyanic acid [13], halogens [150], monochloroacetic acid [151], chloroform [152], water vapor [153], and H_2_ [154]. Another thoroughly investigated conducting polymer is PPy that is sensitive to pH [155,156] hydrogen halide, hydrocyanic acid, 1,3,5-trichloro methylbenzene, methylbenzyl bromide, bromobenzyl cyanide, cyanogen chloride [157], and cyanogen.

#### 5.2.1. Sensors for Organic Compounds

Barisci et al. manufactured numerous sensors with eight PPy-based films on a chip that was doped with eight different doping agents [158]. By means of the resulting eight simultaneous signals, the researchers identified and quantified benzene, toluene, ethyl benzene, and xylol compounds. The same approach was utilized by Hamilton et al. [159], who coated seven sensors with PPy films containing various counteranions (composed of perchlorate, hexafluorophosphate, and sulfonate anions) in different concentrations. The generated materials were used as proof for the existence of five vapors: methanol, ethanol, acetone, butanone, and pentanone. Deng et al. [160] developed eight different conducting polymer films consisting of pyrrole and N-substituted pyrrole derivates. These N-substituted pyrrole derivates were tested as sensitive materials for gas measurements, and results revealed that they are suited to the coating of a large variety of organic vapor measurements (alcohols, triethylamine, hexane, toluene, and acetonitrile) [160].

#### 5.2.2. Biosensors and Hydrogels

Hydrogels are three-dimensional, insoluble, cross-linked, biological fabrics, almost like polymeric network materials, which are able to retain a large amount of water/biological liquids in their swollen network. Conducting polymeric hydrogels may react to physical–chemical transformations as a reaction to changes in ambient conditions, including pH value, temperature, electrical fields, ionic strength, type of salt, solvent, external stresses, light, or a combination thereof. Conducting polymeric hydrogels offer an excellent interface between the phase transporting electrons (electrode) and transporting ions (electrolyte), between natural and synthetic biological systems, and between soft and stiff materials [139]. PAni-based hydrogels have so far been employed for biomedical applications; however, their poor mechanical properties generate tremendous issues in this field.

PAni that are crosslinked with carboxymethyl cellulose (CMC) are processed to form CMC–PAni hydrogels and subsequently impregnated with ammonium persulphate (APS) as oxidizing agent to be used in antimicrobial applications. PAni–Pt biosensors based on nanoparticles were used as proof of human metabolites (metabolism) and manufactured from PAni hydrogel, phytic acid as crosslinker, and platinum (Pt) nanoparticles. Moreover, various enzymes, such as uricase, cholinesterase/cholesterol oxidase, and lipase/glycerol kinase/glycerol-3-phosphate oxidase can be immobilized on this type of hydrogel [161]. A highly sensitive amperometric immunosensor was produced by electrochemical treatment and the use of a carcinoma antigen 125 (CA125) base on a PAni-polythreonine hydrogel [162].

**Figure 24:**
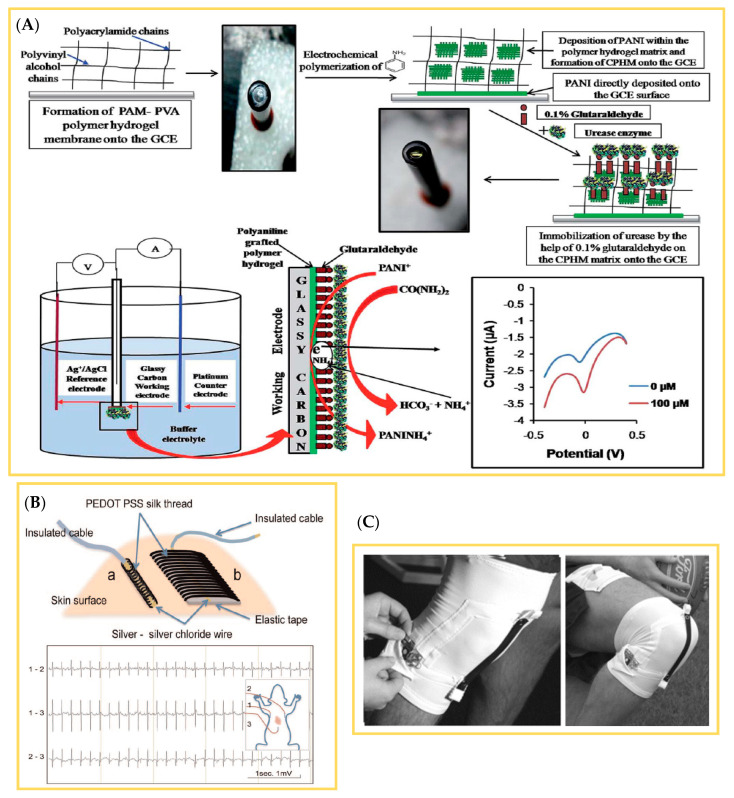
(**A**): Detailed description of the process for the preparation of the conducting polymer hydrogel membrane [166]. (**B**): Electrophysiological recordings obtained using electrodes made with PEDOT:PSS silk glycerol thread. Structure of skin surface electrodes using PEDOT:PSS glycerol silk thread. a. String shaped electrode for electrocardiograph (ECG). b. Flat electrode for electroencephalogram (EEG). ECG recordings obtained from an experimental rat with bipolar deviation. Inset shows the position of the three electrodes [131]. (**C**): Intelligent knee sleeve, with electronics and a conductive fabric strip being worn by an athlete [90].

A polyacrylamide–polyvinyl alcohol hydrogel was utilized as biosensor for the detection of urea (Figure 24A) [163]. Castro et al. [164] synthesized PAni with L-glutamic acid to form a protonation agent, to which amoxicillin and acrylamide solution were added, thus manufacturing a drug delivery hydrogel. Interestingly, hydrogels without PAni exhibited no ON–OFF release pattern, making PAni a descisive factor for successful drug release systems [165]. Orefice et al. introduced an innovative technique for the production of poly(isopropylacrylamide-co-acrylic acid)-PAA–PAni hydrogel through photochemical synthesis using 2-hydroxy-2-methylpropiophenone as photoinitiator. These sensors were designed for actuator applications and are highly sensitive [166]. Takamatsu et al. developed textile-based wearable devices (Figure 9) to record high quality EKG in clinic and ambulatory conditions and to determine heart rate [23]. PEDOT:PSS glycerol silk thread were used for electrocardiograph and for electroencephalogram (Figure 24B) [131]. The intelligent knee sleeve, shown in Figure 24C, has been designed using an ICP-coated textile to monitor and provide direct feedback to the wearer alerting them as to when they have achieved sufficient knee flexion during landing [90].

### 5.3. Protective Coating

Products based on ICPs have been commercially available for a while now; they are currently used in special technical applications, e.g., for corrosion protection and the through-plating/direct metallization of circuit boards [166,167]. PAni is marketed under trade names such as ORMECON or Panipol W. These products even offer metallic properties, so they are often described as “organic metal”. They are mainly employed for the final coating of circuit boards and for corrosion protection. The passivation of metal surfaces is most frequently performed using PAni, however PPy and PT are sometimes used as well. Metals and alloys, e.g., stainless steel, copper, and aluminum, were often selected for biopolar plates [168,169,170]. In recent years, a variety of conducting polymer coatings, including PAni, PT, and PPy, were developed for bipolar plates offering sufficient corrosion protection in addition to high conductivity [167]. For practical applications, the long-term corrosion resistance of conducting polymer coatings must be improved, which is particularly important for protecting metallic bipolar plates in the aggressive environment of Proton Exchange Membrane Fuel Cell (PEMFC). GO was added to the PPPy matrix.

## 6. Conclusions und Outlook

Despite numerous completed research projects, fibers or textile fabrics with high electrical conductivity, excellent strength, and sufficient elongation have not yet been successfully developed. To achieve ICPs with high electrical conductivity and sufficient stability, the corresponding polymers must be fully doped. Doping, oxidation, and reduction processes prior to and after coating, printing, and spinning have so far been neglected by researchers; hence, the relationships between substrate and intrinsically conducting solution have been insufficiently investigated. The most frequently used ICPs for the manufacturing of electrically conducting textile materials are made of natural or chemical fibers, i.e., PEDOT, PAni, and PPy. This review paper gave an overview on different types of ICPs; application and spinning methods for electrically conducting fibers and fabrics based on ICPs; and influencing parameters of coating, printing, or spinning processes affecting the electrical conductivity and stability of electrically conducting layers. Enormous differences in electrical conductivity levels and a large variety of doping, oxidation, binding, and dispersion agents were used for different coating, printing, or spinning methods. With increasing research efforts in the field of ICPs, numerous interesting properties on the textile surface were discovered and developed, such as electrical conductivity, electromagnetic shielding, and antibacterial effects, which must be taken into account in combination with complex requirements to create stable coated textiles. Various coating processes yield different results in terms of layer thickness, morphology, and electrical power of fibers and textiles coated with ICPs. Furthermore, the conducting layer or coated yarn must be resistant to the stresses and strains that occur during textile processing techniques (weaving, knitting, warp knitting, embroidering). Once these polymers are modified for commercial use, they are again subject to processing, including washing, shear loads, and friction forces. In addition, the coating process may induce tensions as well, thus affecting the mechanical properties of the fiber. It is therefore essential that suitable catalysts, doping and oxidizing agents as well as additives are selected for coating, spinning, and printing, which in turn influence the degree of stiffness and resistance of layers.

The limited availability of commercially available ICP products with a variety of concentrations poses a considerable issue for textile companies that are involved in the development of innovative textile applications. Although ICPs are promising materials, their use is currently restricted due to their high costs. Moreover, the majority of investigations was carried out discontinuously; if testing were to focus on continuous, large-scale coating, then additional processing parameters would have to be addressed. Hence, the electrically conducting characteristic of ICPs is combined with the flexibility and stretchability of textile materials. Thus, a completely new functionality and a large amount of potential application scenarios were generated. Based on the resulting conducting fibers and/or textile fabrics, various electronic devices for textiles can be created, for example: physicochemical sensors, thermoelectrical fibers/fabrics, heated clothing, artificial muscles, and textile supercapacitors. Due to their variable properties, the application of ICPs is future-oriented and can be extended beyond the borders of their current niche. However, more research is needed to obtain sufficient data for the prediction of material behavior and integration of this polymer class in new products.

## Figures and Tables

**Table 1:** Electrical conductivity of ICP in a doped an undoped state [1,2,3,4,5].

	Electrical Conductivity (S/cm)						
Polymer	PAc	PPy	PT	PEDOT	PAni	PPV	PPP
**Doped**	10^6^–0^8^	10^2^–10^4^	10^2^	10^2^–300	10^2^	10^2^–10^−3^	10^2^–500
**Undoped**	10^−2^–10^−8^	10^−8^	10^−8^	10^−8^	10^−8^–10^−10^	10^−8^–10^−13^	10^−8^

**Table 2:** Textile materials printed with intrinsically conducting polymers [23,24,25,26,27,28,29,30,31,32].

Material	Method	ICP	Literature
Polyester (PES)	Screen printing	PEDOT:PSS	[23]
PU-PA6	Screen printing	PPy	[24]
PES	Screen printing	PEDOT:PSS	[25]
PES, PA	Screen printing	PEDOT or PAni	[26]
CO	Screen printing	PEDOT:PSS	[27]
PET + elastane + PVF	Screen printing	PEDOT:PSS	[28]
PA, CO	Inkjet printing	PEDOT:PSS	[29,30]
PA	Inkjet printing	PEDOT:PSS	[31,32]

**Table 3:** Electrical properties of fibers.

ICP	Method	Doping Agent/Oxidant	Conductivity (σ) in S/cm	Reference
PAni	Electrospinning	H_2_SO_4_	0.1	[46]
PAni	Electrospinning	H_2_SO_4_ or HCl	52.9	[47]
PAni nanofibers	Electrospinning	PAni (0.5 wt.%)/PMMA(0.54 wt.%) PAni (0.5 wt.%)//PEO(1 wt.%) PAni (100%)	10^−10^ 10^−6^ 50 ± 30	[48]
PAni/PEO bicomponent fibers	Electrospinning	PEO 3/4/5w/v% PAni 1.5/2.5/3.5 w/v%	10^−6^ to 10^−4^	[49]
PAni with PSS, PC, PMMA, and PEO	Electrospinning	PAni/PS PAni/PMMA PAni/PC PAni/PEO	4.1 × 10^−14^ 4.3 × 10^−14^ 5.5 × 10^−14^ 2.4 × 10^−13^	[56]
PEDOT/PSS/PEO nanofibers	Electrospinning	PEDOT:PSS	35.5	[58]
PEDOT/PSS/PVA nanofibers	Electrospinning	PEDOT/PSS/PVA with (0%, 3%, 5%, 8%) of DMSO	4.8 × 10^−8^ 1.7 × 10^−5^	[59]
PPy	Electrospinning	PPy-mwCNT	10^−1^	[60]
P3HT nanofiber	Electrospinning	Iodine	122 ± 9	[63]
PEDOT:PSS	Wet spinning	PEDOT:PSS	10^−1^	[65]
PEDOT:PSS	Wet spinning	PEDOT:PSS/EG	195	[66]
PPy	Wet spinning	PPy/DEHS	3	[73]
PAni with Poly-ω- aminoundecanoic acid	Wet spinning	PAni 5 wt.% PAni 12 wt.% PAni 20 wt.%	10^−5^ 10^−2^ 10^−1^	[74,75]
Silk fiber with PAni	Wet spinning	0.009 wt.% PAni 0.28 wt.% PAni	6.546 × 10^4^ 0.704 × 10^4^	[76]
PAni/co-PAN	Wet spinning	PAni/PAN/DBSA	10^−3^	[78]
PAni/PP	Melt spinning	PAni/PP/DBSA	10^−9^	[82]
PAni/PP matrix fibril fiber	Melt spinning	PAni/PP	10^−4^	[83]
P3HT fiber	Melt spinning	Doping with FeCl_3_	160	[84]

**Table 4:** Textile yarns and fabrics coated with intrinsically conductive polymers.

ICP	Textile Yarns and Fabrics	Coating Technique	Electrical Resistivity or Conductivity	Reference
PAni	WO, CO, PA, and PET yarn	Solution polymerization	23 kΩ/cm/filament	[88]
PPy	PA-PU fabric	Solution polymerization	149 Ω/square	[91]
PPy	PA-PU fabric	Currentless coating	-	[92]
PAni	PES fabric	In-situ polymerization	-	[93]
PT PPy PAni	PET fabric	Knife-over-roll	3.8 × 10^12^ Ω (1.3 wt.%) 2.5 × 10^8^ Ω (5.3 wt.%) 5.9 × 10^12^ Ω (6.2 wt.%)	[95]
PEDOT:PSS/EG	SE yarn	Dipping and drying	8.5 S/cm	[96]
PPy	CO	Dipping and drying	–	[97]
PAni	CO and PET fabric	Dipping and drying	2.28 × 10^−4^ S/cm (CO) 2.15 × 10^−2^ S/cm (PET)	[98]
PEDOT:PSS/EG	PET and PU fabric	Dipping and drying	-	[103]
PPy	WO yarn	Solution polymerization	50 Ω/cm	[104]
PPy	WO, CO, and PA yarn	Vapor polymerization	0.37–3 kΩ/mm	[105]
PEDOT:PSS	E-glass roving	In-situ polymerization	100 kΩ	[107]
PEDOT	PES fiber	Dipping and drying	600 Ω/cm	[108]
PAni	PU yarn	Solution polymerization	10	[112]
PEDOT:PSS	Viscose (CV)	Chemical vapor polymerization	-	[118]
PEDOT:PSS	CV and PET	Chemical vapor deposition	-	[119,120]
PEDOT	CO and PET yarn	Vapor polymerization	10	[126]
PPy	WO yarn	Vapor polymerization	0.43 kΩ/mm	[129]
PPy	CO and SE yarn	Vapor polymerization and solution polymerization	6.4 × 10^−4^ S/cm (CO) and 3.2 × 10^−4^ S/cm (SE)	[130]
PEDOT:PSS	SE yarn	Dipping and drying	2 kΩ/mm	[131]
PAni	PET yarn	Vapor polymerization	3 × 10^5^ Ω/square	[132]
PPy (FC) PPy (FC + NDC)	PET nonwoven	Dipping and drying	178.9 Ω (FC) 100.6 Ω (FC + NDC)	[133,137]
PPy(TiO_2_)	CO fabric	In-situ polymerization	-	[134]
